# miR-346 and miR-138 competitively regulate hTERT in GRSF1- and AGO2-dependent manners, respectively

**DOI:** 10.1038/srep15793

**Published:** 2015-10-28

**Authors:** Ge Song, Renjie Wang, Junfei Guo, Xuyuan Liu, Fang Wang, Ying Qi, Haiying Wan, Min Liu, Xin Li, Hua Tang

**Affiliations:** 1 Tianjin Life Science Research Center, School of Basic Medical Sciences, Tianjin Medical University, Tianjin, China

## Abstract

miRNAs typically downregulate the expression of target genes by binding to their 3′UTR, and dysregulation of miRNAs may contribute to tumorigenesis. Here, we found that miR-346 and miR-138 competitively bind to a common region in the 3′UTR of hTERT mRNA and have opposite effects on the expression and function of hTERT in human cervical cancer cells. Furthermore, G-rich RNA sequence binding factor 1 (GRSF1) mediates the miR-346-dependent upregulation of hTERT by binding to the miR-346 middle sequence motif (CCGCAU) which forms a “bulge loop” when miR-346 is bound to the hTERT 3′UTR, facilitating the recruitment of hTERT mRNA to ribosomes to promote translation in an AGO2-independent manner. Conversely, miR-138 suppresses hTERT expression in an AGO2-dependent manner. Interestingly, replacement of the miR-138 middle sequence with that of miR-346 results in an upregulation of hTERT expression in a GRSF1-dependent manner. Moreover, miR-346 depends on GRSF1 to upregulate another target gene, activin A receptor, type IIB (ACVR2B), in which miR-346 “CCGCAU” motif is essential. These findings reveal novel mechanisms of miRNA-mediated upregulation of target gene expression and describe the coordinated action of multiple miRNAs to control the fate of a single target mRNA through binding to its 3′UTR.

miRNAs are a class of endogenously conserved small RNAs that regulate target gene expression by affecting mRNA translation and stability or by modulating the promoter activity of target genes^1^,^2^. miRNAs may control the majority of human genes, which are involved in a wide variety of biological and pathogenic processes, including cancers^3^,^4^,^5^,^6^. Dysregulation of miRNAs in cancer cells influences the function of oncogenes and tumor suppressors^5^,^7^. In most cases, miRNAs suppress the expression of target genes by binding to their 3′UTR. Conversely, some miRNAs have been reported to activate the expression of target genes by different mechanisms, including relief of target gene silencing and direct activation of target gene^8^,^9^,^10^. For example, miR-369–3p mediates the upregulation of TNFα translation through AU-rich elements (AREs) under serum-starved conditions^11^. miR-373 has been revealed to upregulate E-cadherin and cold-shock domain-containing protein C2 (CSDC2) expression through complementarily binding to their promoters^12^. In addition, we recently found that miR-20a and miR-490-3p bind the tankyrase 2 (TNKS2) and the endoplasmic reticulum-Golgi intermediate compartment protein 3 (ERGIC3) 3′UTRs, respectively, to upregulate their expression^13^,^14^. There is also evidence that multiple miRNAs target the same region in the 3′UTRs to regulate target genes expression^15^. For instance, miR-15a and miR-16 can both suppress BCL2 expression by binding the same region of its 3′UTR^16^. However, it is unclear how miRNAs upregulate target genes expression by association with their 3′UTRs and whether a single mRNA can be targeted by multiple miRNAs that exert opposing effects on gene expression through binding to a common site in the target gene′s 3′UTR.

The human telomerase reverse transcriptase (hTERT) is an essential subunit of human telomerase, which modulates the expression of growth-controlling genes and enhances cell proliferation^17^,^18^,^19^. Although hTERT is undetectable in normal somatic tissues, it is activated in embryogenesis and approximately 85% of human cancers^17^,^20^. The expression of hTERT is regulated through complex transcriptional and post-transcriptional mechanisms. Some transcription factors, especially c-Myc and β-catenin^21^,^22^, and epigenetic modifications in hTERT promoter region, including histone acetylation and methylation, participate in the regulation of hTERT expression^20^. The activity of hTERT is also regulated through phosphorylation by Akt kinase and modulation by nuclear factor-κB p65^23^. However, the role of miRNAs in the regulation of hTERT is not well understood.

GRSF1, a member of the hnRNP F/H RNA binding protein family involving in RNA splicing, RNA stability, RNA capping and translation^24^, is reported to bind cellular and viral mRNAs, such as the influenza virus nucleoprotein (NP) gene mRNA, and recruits them to polyribosomes for translation^25^,^26^,^27^. In addition, GRSF1 accumulates in the mitochondrial matrix to regulate mitochondrial nascent RNA processing^28^,^29^. However, whether GRSF1 is involved in miR-mediated gene expression remains to be determined.

In this study, we found that miR-346 is upregulated and that miR-138 is downregulated in human cervical cancer tissues. The middle sequence motif (nt 8–13, CCGCAU) of miR-346 is required to enhance hTERT expression through a GRSF1-mediated recruitment of hTERT mRNA to polysomes for translation in an AGO2-independent manner. In contrast, miR-138 has been shown to downregulate the expression of hTERT in AGO2-dependent manner. Despite the opposite effects on hTERT expression, the miR-346 and miR-138 binding sites are in a common region of the hTERT 3′UTR, in which the coordinated regulation results in the upregulation of hTERT and contributes to the growth of cervical cancer cells. The critical roles of miR-346 “CCGCAU” motif and GRSF1 are further confirmed through the studies of activin A receptor, type IIB (ACVR2B), SMAD family member 3 (SMAD3) and miR-138 middle sequence motif. These findings shed light on miRNA-mediated upregulation of target mRNAs through 3′UTR binding and provide novel insight into the mechanisms by which multiple miRNAs modulate the expression of a single mRNA.

## Results

### miR-346 promotes the growth of human cervical cancer cells and upregulates hTERT expression by targeting its 3′UTR

**Figure 1:**
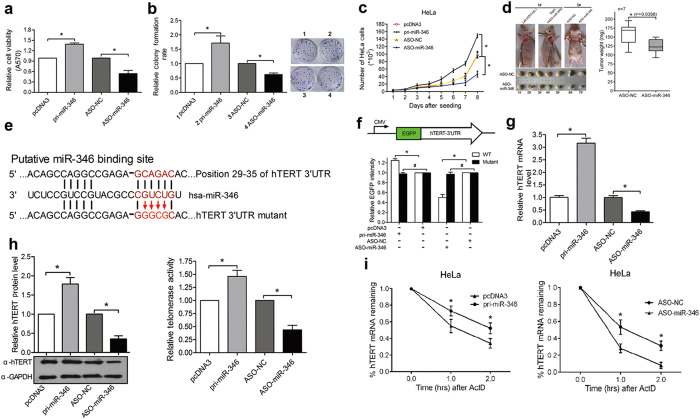
miR-346 enhances the growth of human cervical cancer cells by directly targeting the 3′UTR of hTERT mRNA both *in vitro* and *in vivo.* (**a–c**) MTT assay (**a**), colony formation assay (**b**) and growth curve assays (**c**) were performed to evaluate the cell viability and growth capacity of HeLa cells transfected with pri-miR-346 or ASO-miR-346 and their respective controls. The data from the treated groups were normalized to the data of the control groups. (**d**) ASO-miR-346 treatment overtly repressed the growth of xenograft cervical cancers derived from HeLa cells in SCID mice (n = 7). (**e**) The predicted miR-346 binding site and the point mutation within the “seed region” binding site on the hTERT mRNA 3′UTR are shown. (**f**) EGFP reporter assays were performed to determine the effects of miR-346 on the hTERT 3′UTR. HeLa cells were transfected with the wild type EGFP-hTERT 3′UTR (WT) or mutated EGFP-hTERT 3′UTR (mutant) reporter vectors and either ASO-miR-346, pri-miR-346, control ASO or the control vector. (**g,h**) The influence of miR-346 on endogenous hTERT expression was evaluated by qRT-PCR (**g**) and western blot (h, left panel). The blot was cropped and the full-length blot is presented in Supplementary Fig. S8. The levels of hTERT mRNA and protein were normalized to the levels of β-actin mRNA and GAPDH protein, respectively. The telomerase activity in HeLa cells transfected with pri-miR-346 or ASO-miR-346 was evaluated using the *T*elo*TAGGG* Telomerase PCR ELISA^PLUS^ kit (Roche) (h, right panel). (**i**) miR-346 affects the stability of hTERT mRNA. The amount of remaining hTERT mRNA 1 and 2 hr after ActD (5 μg/ml) treatment were measured by qRT-PCR and normalized to the hTERT expression levels at 0 hr in HeLa cells. *p < 0.05, ^#^p > 0.05.

To determine the role of miR-346 in the growth of cervical cancer cells, the ectopic expression plasmid of miR-346 (pri-miR-346) and antisense oligomers (ASO-miR-346) were used to overexpress or block miR-346, respectively, in HeLa and C33A human cervical cancer cell lines (Fig. S1a,b). It was found that pri-miR-346 promoted, whereas ASO-miR-346 suppressed cell viability and cell growth by MTT, colony formation and growth curve assays compared to the control groups in both cell lines (Fig. 1a–c and S2a–c). Furthermore, a xenograft tumor model was used to determine whether miR-346 is involved in tumorigenesis *in vivo* in a loss-of-function manner. HeLa cells transfected with ASO-miR-346 or ASO-NC were injected into severe combined immunodeficiency (SCID) mice. As a result, the average weight of tumors derived from ASO-miR-346-treated HeLa cells was reduced by approximately 30% compared with that of the control group (Fig. 1d). These results confirm that miR-346 facilitates cervical cancer cell growth.

To identify the target genes of miR-346 that promote growth in cervical cancer cells, bioinformatics analysis showed that hTERT contains a putative miR-346 binding site in its 3′UTR (Fig. 1e). Then an enhanced green fluorescence protein (EGFP) reporter assay was used to validate whether miR-346 directly binds the 3′UTR of hTERT mRNA. hTERT 3′UTR fragment containing either the predicted miR-346 binding site or a mutant binding site (Fig. 1e) was inserted downstream of the EGFP gene following a stop codon (pEGFP-hTERT 3′UTR, pEGFP-hTERT 3′UTR mut). HeLa cells were transfected with this reporter vector along with pri-miR-346, a control vector, ASO-miR-346 or the ASO-NC. pri-miR-346 increased the intensity of the EGFP fluorescence, whereas ASO-miR-346 significantly decreased it compared with the control groups (Fig. 1f). In contrast, the expression of the 3′UTR mutant reporter was not affected by the alteration of miR-346 level (Fig. 1f). These results indicate that hTERT mRNA is a direct target of miR-346. Meanwhile, the pri-miR-346 increased endogenous hTERT mRNA and protein level more than 2 fold, but ASO-miR-346 decreased it by approximately 45% compared with the control groups (Fig. 1g,h, left panel). Likewise, telomere repeat amplification protocol (TRAP) analysis showed that pri-miR-346 significantly enhanced, but ASO-miR-346 reduced the telomerase activity (Fig. 1h, right panel and Fig. S8). Furthermore, we used actinomycin D (ActD) that blocks the synthesis of new transcripts to analyze hTERT mRNA stability in HeLa cells. As shown in Fig. 1i, pri-miR-346 increased, whereas ASO-miR-346 decreased the stability of hTERT mRNA following treatment with ActD. These results indicate that miR-346 enhances hTERT expression and activity and may protect hTERT mRNA from degradation.

A previous study has shown that miR-138 downregulates the expression of hTERT in human anaplastic thyroid carcinoma cell lines^30^. We also demonstrated that miR-138 targets the hTERT 3′UTR, suppressing its expression and inhibiting HeLa cell growth (Fig. S1d,e and S3a–h). Thus, ectopic expression of miR-138 and inhibition of miR-346 using ASO were used for further studies.

### Restoration of hTERT expression abrogates the growth suppression of HeLa cells caused by miR-346 inhibition or miR-138 overexpression

**Figure 2:**
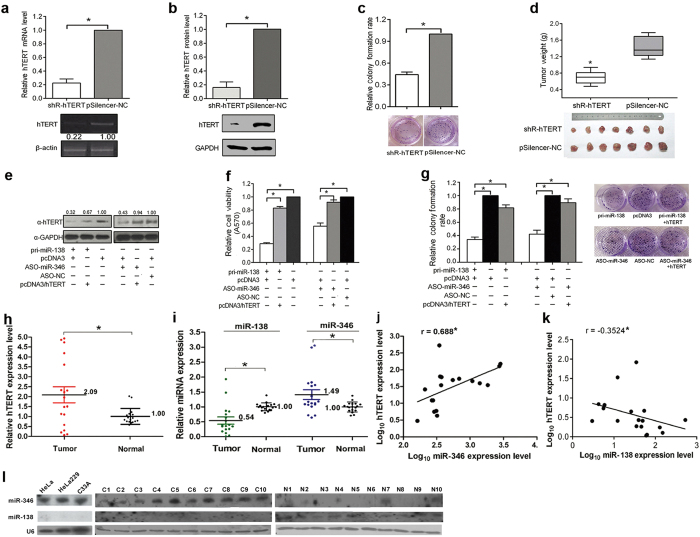
hTERT is a functional target of both miR-346 and miR-138. (**a,b**) The efficiency of shR-hTERT was confirmed by qRT-PCR and western blot assays. (**c**) The suppressive effects of shR-hTERT on HeLa cell growth were evaluated by colony formation assays. The histograms show the normalized colony formation rate ± SD from three independent experiments. *p < 0.05. (**d**) The growth of tumors derived from HeLa cells treated with shR-hTERT or pSilencer-NC was evaluated. The data are shown as the mean ± SD of 7 mice from each group. *p < 0.05. (**e–g**) hTERT overexpression plasmid without the 3′UTR, pcDNA3/hTERT, and ASO-miR-346 or pri-miR-138 were transfected into HeLa cells, with pcDNA3 and ASO-NC used as negative controls. The expression of hTERT protein was detected by western blot (**e**). Overexpression of hTERT counteracts the cell viability (**f**) and growth inhibition (**g**) caused by ectopic miR-138 or ASO-miR-346 expression in HeLa cells. The data from the treated groups were normalized to the control group. (**h,i**) The expression levels of hTERT mRNA, miR-346 and miR-138 were assessed by qRT-PCR in 18 pairs of cervical cancer tissues and their matched normal tissues. The means of RNA expression were determined using the formula 2^−(ΔΔCT)^, where ΔΔCT = [Ct (tumor-mRNA) - Ct (tumor-control)] - [Ct (non-tumor-mRNA) - Ct (non-tumor-control)], and normalized to β-actin mRNA and U6 RNA for the mRNAs and miRNAs, respectively. The RNA expression levels of cervical cancer tissues were normalized to normal tissues, whereas the RNA expression levels of the matched normal tissues were compared to the average level of all normal tissues. (**j,k**) The correlations between miR-346 (r = 0.688) or miR-138 (r = −0.3524) and hTERT mRNA were calculated using the Pearson correlation test with the software of GraphPad Prism 6.01. (**l**) miR-346 and miR-138 in three cervical cell lines and 10 pairs of matched cervical cancer and normal tissues were assessed by Northern blot. The blots and gels were cropped and the full-length blots/gels are presented in Supplementary Fig. S8.

To determine the effect of hTERT on the growth of HeLa cells, hTERT was knocked down using a small hairpin RNA expression plasmid (shR-hTERT) (Fig. 2a,b, Fig. S8). shR-hTERT markedly reduced HeLa cell growth *in vitro* and *in vivo* assessed by colony formation assay and a xenograft tumor model (Fig. 2c,d). These results indicate that hTERT promotes the growth of HeLa cells.

To validate that miR-346 promotes and miR-138 suppresses the growth of HeLa cells by directly upregulating hTERT, the “rescue” experiments were performed using an hTERT expression vector (pcDNA3/hTERT) containing the hTERT ORF without the 3′UTR (Fig. S4a,b and Fig. S10). When HeLa cells were cotransfected with ASO-miR-346 or pri-miR-138 and pcDNA3/hTERT, the inhibition of hTERT expression, cell viability and the colony formation rate were rescued by hTERT expression (Fig. 2e–g and Fig. S8). These results indicate that hTERT is a common mediator of miR-346-activated and miR-138-suppressed cell growth in HeLa cells.

In addition, compared to the adjacent normal cervical tissues, the increased levels of hTERT mRNA as well as miR-346 and decreased levels of miR-138 were found by qRT-PCR in eighteen human cervical cancer tissues (Fig. 2h,i), and the aberrant expression of both miRNAs were futher confirmed by northern blot assays in both cervical cancer tissues and cervical cancer cell lines (Fig. 2l and S8). Meanwhile, a positive correlation between the hTERT and miR-346 expression (Fig. 2j), but a negative correlation between the hTERT and miR-138 (Fig. 2k) was observed, indicating that enhanced hTERT expression correlate with increased miR-346 and decreased miR-138 in human cervical cancer tissues and cell lines.

### miR-346 and miR-138 bind a common site in the hTERT 3′UTR to coordinately regulate its expression

**Figure 3:**
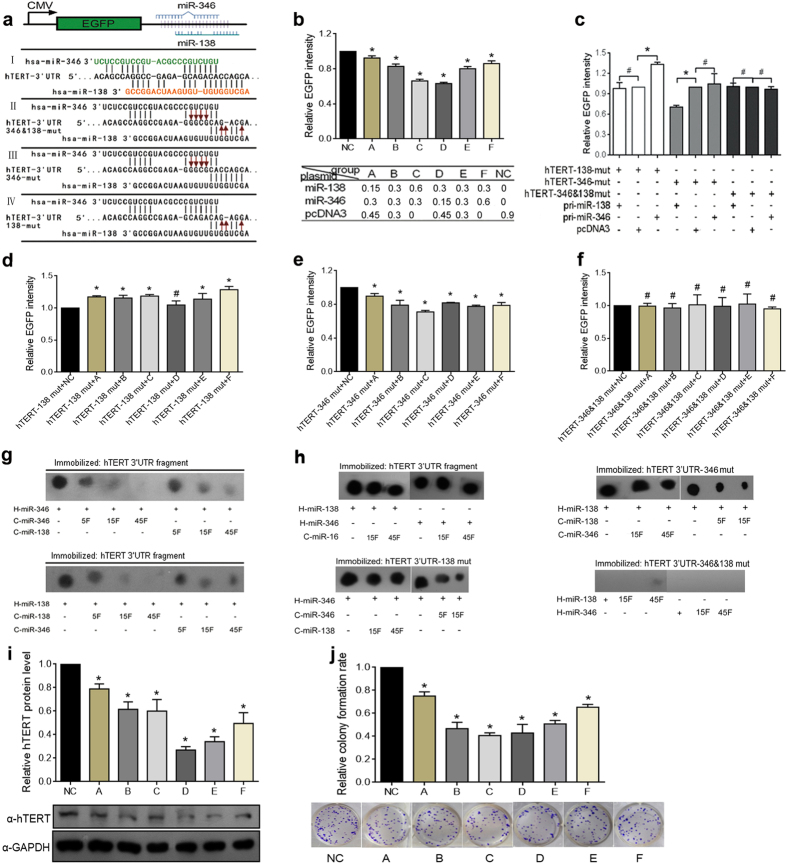
miR-346 and miR-138 competitively regulate hTERT expression and function. (**a**) (I) The predicted miR-346 and miR-138 binding sites on the hTERT mRNA 3′UTR, (II) a double mutant of both the miR-346 and miR-138 binding sites, and (III) miR-346 or (IV) miR-138 single mutant binding sites. (**b**) EGFP reporter assays were performed to evaluate the effects of various concentrations of miR-346 to miR-138 ratios on wild type hTERT 3′UTRs. The EFGP intensity was normalized to RFP. The present doses of plasmids are for 24-well plastic cell culture plate. (**c**) EGFP reporter assays were used to evaluate the effects of miR-346 and miR-138 on the three types of mutant hTERT 3′UTR. The relative EGFP/RFP intensity in treated group was normalized to the control group. (**d–f**) EGFP reporter assays were adopted to analyze the influence of varied ratios of miR-346 to miR-138 on EGFP reporters with the three mutant forms of the hTERT 3′UTR. The EFGP intensity was normalized to control group. (**g,h**) Dot blot hybridization assays show dose-dependent competition between miR-346 and miR-138 for binding to the wild type hTERT 3′UTR fragment and three types of mutant hTERT 3′UTRs. miR-16 is a non-competing miRNA and serves as a negative control. H and C represent hot (labeled) and cold (non-labeled) miRNAs. The blots represented the full-length blots. (**i**) The relative hTERT protein levels in HeLa cells transfected with different ratios of miR-346 to miR-138 were evaluated by western blot. GAPDH was used as a loading control. The gels were cropped and the full-length gels are presented in Supplementary Fig. S8. (**j**) The colony formation rate increased with an increasing miR-346 to miR-138 ratio. The experiments were performed in triplicate. *p < 0.05, ^#^p > 0.05.

Bioinformatics analysis showed that miR-138 and miR-346 target common regions in the hTERT 3′UTR that overlap by 15 nucleotides (nt 21–40 and 16–35, respectively). The miR-138 and miR-346 seed sequences are complementary to nt 34–40 and nt 29–35 of the hTERT 3′UTR, respectively; the 9 bases that mediate miR-346 binding are the same as those that mediate the miR-138 interaction (Fig. 3a-I). This observation prompted us to explore whether miR-346 and miR-138 competitively regulate hTERT expression.

To address this question, an EGFP reporter vector containing an hTERT 3′UTR fragment with miR-346 and miR-138 binding sites was transfected along with various concentrations of pri-miR-346, pri-miR-138 or a control vector into HeLa cells (Fig. 3b, bottom panel). And there was a positive correlation between the EGFP expression levels and the ratio of pri-miR-346 to pri-miR-138 in the context of a fixed concentration of miR-346 or miR-138 (Fig. 3b). However, the EGFP intensity for the cells transfected with pcDNA3/EGFP containing a mutated miRNA binding site (138-mut or 346-mut) (Fig. 3a-II, -III, -IV) was not influenced by itself, but by the other miRNA (Fig. 3c). Mutation of both binding sites (346- & 138-mut) abolished the regulation of EGFP expression by both miR-138 and miR-346 (Fig. 3c). Furthermore, the EGFP intensities in HeLa cells transfected with the 138-mut or 346-mut reporter vector were enhanced with increased miR-346 (Fig. 3d) or decreased miR-138 (Fig. 3e); however, mutation of both the miR-138 and miR-346 binding sites abolished regulation whatever the ratio of miR-346 to miR-138 was (Fig. 3f). Moreover, we used a dot blot hybridization assay to confirm the competitive binding, in which ^32^P-labeled miR-346 or miR-138 (hot miR-346, hot-miR-138) served as a probe and the non-labeled miR-16 (cold miR-16) as a negative control for competing experiment of miR-346 and miR-138 binding hTERT 3′UTR. As shown in Fig. 3g, increasing the levels of cold miR-138 decreased the binding of hot miR-346 to the hTERT 3′UTR fragment, and vice versa. And cold miR-16 did not decrease the binding of hot miR-346 or hot miR-138 to the hTERT 3′UTR fragment (Fig. 3h, left panel up). Furthermore, the binding to the hTERT 3′UTR with miR-138 or miR-346 mutant binding sites was weakened by increasing cold miR-346 or cold miR-138, not by itself (Fig. 3h, left panel bottom and right panel up). Whereas the hTERT 3′UTR with 346 & 138 mutant which mutated both miR-138 and miR-346 binding sites was totally abolished to bind to miR-138 and miR-346 (Fig. 3h, right panel bottom). Additionally, the endogenous hTERT protein and colony formation rate both positively correlated with the ratio of pri-miR-346 to pri-miR-138 (Fig. 3i,j and Fig. S8). These results indicate that miR-346 and miR-138 compete for binding to a common region on the hTERT 3′UTR and competitively regulate hTERT expression and function, which depends on the ratio of miR-346 to miR-138.

### miR-346 upregulates hTERT expression in an AGO2-independent manner and requires the miR-346 middle sequence motif

**Figure 4:**
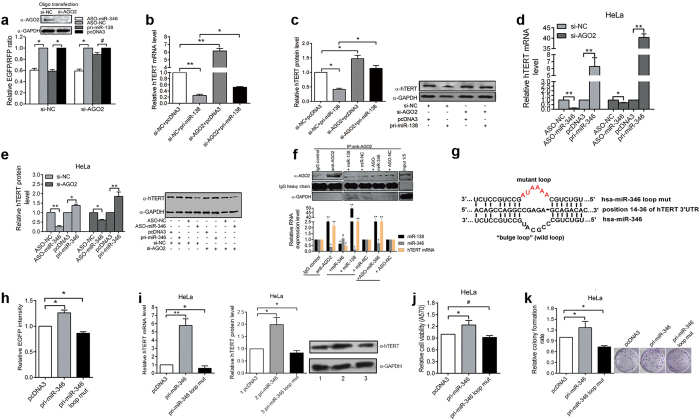
miR-346 upregulates hTERT expression in an AGO2-independent manner, but requires the miR-346 middle sequence motif. (**a**) The efficiency of si-AGO2 was confirmed by western blot (top panel). In EGFP-hTERT 3′UTR reporter assays, AGO2 depletion in HeLa cells abolished miR-138-mediated repression but did not affect the regulation by ASO-miR-346 (bottom panel). (**b–e**) Knockdown of AGO2 with synthesized siRNA (25 pmol/6-well plastic culture plate for RNA isolation; 5 pmol/24-well plastic culture plate for protein lysis) in HeLa cells followed by qRT-PCR and western blot analyses shows the effects of pri-miR-138, pri-miR-346 and ASO-miR-346 on endogenous hTERT expression. (**f**) Western blot shows the pull-down of AGO2 protein (top panel), and qRT-PCR shows the levels of miR-138, miR-346 and hTERT mRNA that immunoprecipitated with the AGO2 complex in HeLa cells transfected with miR-138, miR-346 and ASO-miR-346 (bottom panel). (**g**) The diagram shows the “bulge loop” motif formed between miR-346 and the hTERT mRNA 3′UTR. The sequences of the wild type (WT) and mutant miR-346 loops are shown. (**h**) Mutation of the miR-346 middle sequence abolished miR-346-mediated upregulation of EGFP intensity. (**i**) A loop mutation abolished the effect of miR-346 on endogenous hTERT as detected by qRT-PCR and Western blot assays. (**j,k**) MTT and colony formation assays assessed the impact of the loop-mutant miR-346 on HeLa cell viability and growth capacity. The blots were cropped and the full-length gels are presented in Supplementary Fig. S8 and S9. *p < 0.05, **p < 0.01, ^#^p > 0.05.

Since AGO2 is the core effector of RISC, we investigated whether AGO2 is involved in the miR-138- and miR-346-mediated regulation of hTERT expression by RNA interference. Surprisingly, AGO2 depletion abolished the repression of the reporter by miR-138, but did not affect the reporter expression by the ASO-miR-346 compared with the control groups (Fig. 4a and Fig. S8). Similarly, AGO2 knockdown attenuated miR-138-mediated downregulation of endogenous hTERT mRNA and protein expression (Fig. 4b,c, Fig. S9), but had no effects on the ability of either pri-miR-346 or ASO-miR-346 to modulate hTERT expression (Fig. 4d,e, Fig. S8). Furthermore, RNA immunoprecipitation (RIP) analysis showed that miR-138 and hTERT mRNA, but least miR-346 were bound to the AGO2 complex. Overexpression of miR-346 reduced the abundance of hTERT mRNA associated with AGO2, probablely due to more hTERT mRNA bound to miR-346 (Fig. 4f). In addition, depletion of AGO2 reduced miR-138 and hTERT mRNA assembled to AGO2 complex and increased hTERT mRNA bound to miR-346 to GRSF1 complex (Fig. S6a). These results indicate that miR-346-mediated upregulation of hTERT does not require AGO2; in contrast, miR-138-mediated suppression of hTERT expression is AGO2-dependent.

Sequence motifs within a specific miRNA are crucial for its functions. To explore whether a sequence motif within miR-346 is involved in hTERT upregulation, we applied bioinformatics analysis (RNAhybrid) and found that nucleotides (nt) 1 to 7 and 14 to 18 of miR-346 are complementary to nt 35-29 and 23-19 of the hTERT 3′UTR, respectively. The middle sequence of miR-346, nt 8-13 (CCGCAU, 6 nt), does not match nt 28 to 24 (AGAGC, 5 nts) of the hTERT 3′UTR, which causes a “bulge loop” (termed miR-346 loop) (Fig. 4g). To determine whether the miR-346 loop (Fig. S1c) is involved in regulating hTERT expression, firstly, we confirmed that the mutant miR-346 was still capable of binding to hTERT 3′UTR through the dot blot hybridization assay (Fig. S6b). Next, we found that pri-miR-346 loop mut abolished the miR-346-mediated activation of reporter expression, hTERT mRNA and protein expression (Fig. 4h,i, Fig. S9) as well as the promotion of cell viability and colony formation rate (Fig. 4j,k), indicating that the middle sequence motif (nt 8-13, CCGCAU) of miR-346 is crucial for enhancing hTERT expression.

To further illustrate the positive regulation of miR-346 on hTERT by the middle sequence motif of miR-346, a series of experiments were conducted in another cervical cell line C33A. Firstly, the EGFP reporter assay revealed that miR-346 also targeted and upregulated hTERT and pri-miR-346 loop mut abolished its upregulation (Fig. S7a,b and Fig. S10). Simultaneously, pri-miR-346 promoted and ASO-miR-346 suppressed endogenous hTERT mRNA and protein expression, whereas pri-miR-346 loop mut abrogated the promoting effect of miR-346 on hTERT expression (Fig. S7c,d and Fig. S10). Taken together, these data consolidate the positive impacts of miR-346 on hTERT by its “CCGCAU” motif.

**Figure 5:**
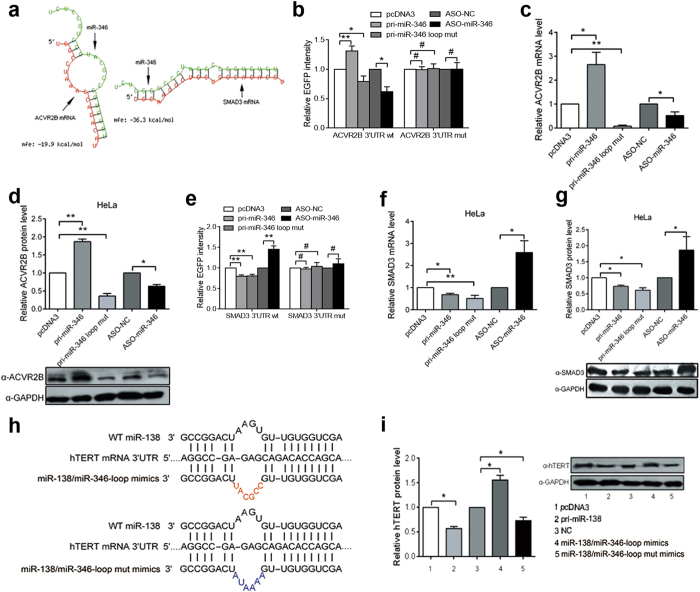
The influences of miR-346 on another two target genes, ACVR2B and SMAD3. (**a**) The secondary structures of the miR-346/ACVR2B 3′TUR and miR-346/SMAD3 3′UTR fragments duplexes were determined using the RNAhybrid algorithm. (**b,e**) The EGFP reporter assays were performed to evaluate the effects of miR-346 on reporter with wt and mutant ACVR2B 3′UTR (**b**) or SMAD3 3′UTR (**e**) with HeLa cells transfected with pri-miR-346, pri-miR-346 loop mut, ASO-miR-346 or the respective controls. The EFGP intensity was normalized to RFP. (**c,d,f,g**) qRT-PCR and western blot were used to detect the endogenous mRNA and protein expression levels of ACVR2B (**c,d**) and SMAD3 (**f,g**) in transfected HeLa cells. β-actin and GAPDH are the loading controls for the mRNA and protein, respectively. (**h**) miR-138/346-loop mimics: synthesized miR-138 with sequence motif of wild type miR-346; miR-138/miR-346-loop mut mimics: synthesized miR-138 with mutant sequence motif of miR-346. The calculated secondary structures of the wild type miR-138, miR-138/346-loop mimics or miR-138/miR-346-loop mut mimics and hTERT 3′UTR duplexes are shown. (**g**) Western blot was used to analyze hTERT protein expression in HeLa cells treated with wild type miR-138, miR-138/346-loop mimics or miR-138/miR-346-loop mut mimics. GAPDH was used as a loading control. The blots were cropped and the full-length blots are presented in Supplementary Fig. S9. *p < 0.05, **p < 0.01.

To further confirm the role of miR-346 “CCGCAU” motif, TargetScanHuman 6.2 and RNAhybrid algorithm were used to predict target genes of miR-346 and analyze the secondary structures of miR-346/target gene mRNAs duplexes, as a result, ACVR2B and SMAD3 were chosed as two different types of target genes: with and without miR-346 loop (Fig. 5a). As expected, the EGFP reporter assay showed that miR-346 targeted and positively regulated ACVR2B expression (Fig. 5b). And pri-miR-346 promoted and ASO-miR-346 suppressed endogenous ACVR2B mRNA and protein expression, while the pri-miR-346 loop mut abolished the activation effects on their expression (Fig. 5c,d, Fig. S9). Conversely, pri-miR-346 decreased and ASO-miR-346 increased the EGFP reporter intensities and endogenous SMAD3 mRNA and protein expression; however, the pri-miR-346 loop mut did not affect the miR-346-mediated suppression (Fig. 5e–g and Fig. S9). Furthermore, RNAhybrid was also used to predict the secondary structure of the miR-138/hTERT 3′UTR duplex. As shown in Fig. 5h, a small and different (UGAA) motif was formed by the miR-138 middle sequence. To further investigate whether replacing the miR-138 motif with the miR-346 motif promotes hTERT expression, we detected endogenous hTERT protein expression by western blot in HeLa cells transfected with synthesized miR-138/346-loop mimics (with the wild type miR-346 “CCGCAU” sequence motif), miR-138/miR-346-loop mut mimics (with mutant sequences of the miR-346 motif) and NC. As expected, the miR-138/346-loop mimics upregulated hTERT protein expression and the miR-138/miR-346-loop mut mimics reversed the promotion of translation (Fig. 5i and Fig. S9). Taken together, these data suggested that the miR-346 middle sequence motif is essential for mediating the upregulation of target genes, which may be universal.

### The middle CCGCAU motif within miR-346 is essential for GRSF1 binding, which is involved in the miR-346-mediated enhancement of hTERT expression

**Figure 6:**
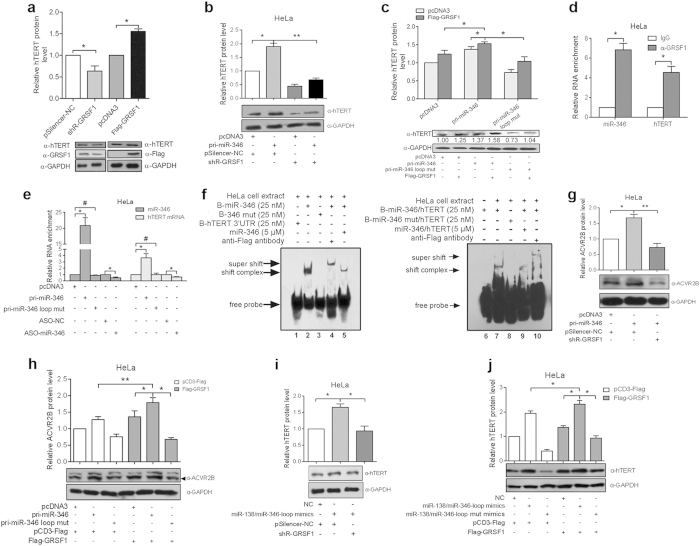
The miR-346 “CCGCAU” motif is crucial for GRSF1 binding. (**a**) Western blot assay showed the expression of hTERT protein in HeLa cells with overexpression or depletion of GRSF1. GAPDH was used as a loading control. (**b**) Western blot assay was used to perform the “rescue” experiment to analyze whether miR-346 upregulates hTERT in a GRSF1-dependent manner. (**c**) Western blot assay showed whether GRSF1 requires the miR-346 sequence motif to mediate the enhancement of hTERT expression in HeLa cells co-transfected with Flag-GRSF1 and pri-miR-346, pri-miR-346 loop mut or control plasmids. GAPDH serves as a loading control. (**d**) The levels of hTERT mRNA and miR-346 that immunoprecipitated with the endogenous GRSF1 complex were detected by qRT-PCR in mock HeLa cells. (**e**) RIP assays using anti-GRSF1 antibody showed that the level of hTERT mRNA in the GRSF1 immunoprecipitation complexes correlated well with the levels of miR-346 in HeLa cells transfected with pri-miR-346, pri-miR-346 loop mut or the control vector and ASO-miR-346 or ASO-NC. (**f**) RNA EMSA was performed with different single-strands RNAs (B-hTERT 3′UTR, B-miR-346 and B-miR-346 mut) or annealed duplexes (B-miR-346/hTERT and B-miR-346 mut/hTERT) and extracts from HeLa cells overexpressing Flag-GRSF1. B: biotin-conjugated. A 200-fold excess of unconjugated miR-346 or miR-346/hTERT served as a competitor. The gels are the full-length gels. (**g**) Western blot was used to detect whether miR-346-mediated upregulation of ACVR2B depends on GRSF1 in HeLa cells co-transfected with miR-346 and shR-GRSF1 or pSilencer-NC. GAPDH acts as a loading control. (**h**) Co-expression of the pri-miR-346 loop mut and Flag-GRSF1 abrogated the upregulation of ACVR2B protein induced by pri-miR-346 and GRSF1. (**i**) Western blot was used to detect whether miR-138/miR-346-loop mimics-mediated upregulation of hTERT depends on GRSF1 in HeLa cells co-transfected with miR-138/miR-346-loop mimics and shR-GRSF1 or pSilencer-NC. (**j**) Co-expression of the miR-138/miR-346-loop mut mimics and Flag-GRSF1 abrogated the upregulation of hTERT protein induced by miR-138/miR-346-loop mimics and GRSF1. The Western blot gels were cropped and the full-length gels are presented in Supplementary Fig. S9 and S10. *p < 0.05, **p < 0.01, ^#^p > 0.05.

Proteins that recognize sequence motifs may control miRNA localization or stability^1^. We noticed that GRSF1 interacts with a G-rich element in mRNAs and mediates the translational enhancement of some cellular and viral mRNAs by recruiting bound mRNAs to polyribosomes^24^. As previously described, the “bulge loop” of miR-346 (CCGCAU) is crucial to hTERT upregulation. Therefore, we speculated that the “CCGCAU” motif might be involved in binding to GRSF1 to promote hTERT expression. To test this hypothesis, we first examined whether changes in GRSF1 levels affected hTERT expression. Overexpression of Flag-tagged GRSF1 (Flag-GRSF1) increased hTERT expression, while knock down of GRSF1 decreased its expression (Fig. 6a and Fig. S9). Co-transfection of miR-346 and shR-GRSF1 led to an approximately 60% reduction in hTERT protein compared with the group co-transfected with miR-346 and the pSilencer-NC (Fig. 6b), indicating that GRSF1 appears to contribute to miR-346-mediated upregulation of hTERT. The similar results were observed in C33A cell line (Fig. S7e and S10). Next, we investigated whether the GRSF1-mediated promotion of hTERT translation was dependent on the miR-346 “CCGCAU” motif. Similarly, co-expression of miR-346 and Flag-GRSF1 led to higher levels of hTERT expression than that the group co-expressing both the miR-346 loop mut and Flag-GRSF1 (Fig. 6c and Fig. S9), demonstrating that GRSF1 promotes hTERT translation in a miR-346-dependent and sequence motif-specific manner. To determine whether GRSF1 forms a complex with miR-346 and hTERT mRNA, we performed a RIP experiment using an anti-GRSF1 antibody in mock HeLa cells. qRT-PCR showed significant enrichment of miR-346 and hTERT mRNA with the precipitated endogenous GRSF1 complexes (Fig. 6d). Moreover, overexpression of miR-346 led to an approximately 4-fold and 20-fold increase, whereas ASO-miR-346 led to a 60% and 40% decrease in the levels of hTERT mRNA and miR-346 associated with the GRSF1 complex, respectively, compared to their controls in transfected HeLa cells by RIP assays. But the miR-346 loop mut did not affect the levels of GRSF1-associated hTERT mRNA compared to control group (Fig. 6e), indicating that GRSF1 could interact with miR-346 and hTERT mRNA. To further confirm the interaction, RNA electrophoretic mobility shift assays (EMSA) were performed. No shift bands were observed in the absence of extracts prepared from HeLa cells expressing Flag-GRSF1 (Fig. 6f, lane 6); the biotin-conjugated hTERT 3′UTR alone also failed to promote a shift band (Fig. 6f, lane 1). However, biotin-conjugated miR-346 and biotin-conjugated miR-346/hTERT 3′UTR fragment annealed duplex generated obviously shifted complexes (Fig. 6f, lane 2 and 7), while the biotin-miR-346 loop mutant (B-miR-346 mut) or B-miR-346 loop mutant /hTERT 3′UTR fragment duplex did not generate a shift band (Fig. 6f, lanes 3 and 8). The unconjugated miR-346 or unconjugated miR-346/hTERT 3′UTR fragment duplexes could compete with their labeled counterparts for binding (Fig. 6f, lane 5 and 9). Addition of an anti-Flag antibody form a super shit band (Fig. 6f, lanes 4 and 10), indicating that the observed interaction is specific and that Flag-GRSF1 is indeed a component of the shifted band. Together, these data indicate that miR-346 alone and that a miR-346/hTERT 3′UTR fragment (annealed duplex) can bind the GRSF1 complex through the miR-346 “CCGCAU” motif, which is involved in miR-346-mediated promotion of hTERT expression.

The above results in Fig. 5 showed that miR-346 and miR-138/346-loop mimics depended on miR-346 “CCGCAU” motif to enhance the expression of ACVR2B and hTERT, respectively. Thus, we investigated whether the promotion requires GRSF1. Co-expression of miR-346 and shR-GRSF1 attenuated the upregulation of ACVR2B protein levels induced by co-expression of miR-346 and pSilencer-NC (Fig. 6g and Fig. S10). Next, we examined whether the GRSF1-mediated upregulation was dependent upon the miR-346 loop motif. We performed western blot to analyze the ACVR2B protein expression following the co-transfection of miR-346 or miR-346 loop mut and Flag-GRSF1 in HeLa cells. As shown in Fig. 6h, co-transfection of miR-346 loop mut and Flag-GRSF1 abolished the miR-346-mediated promotion. Similarly, in co-expression group, shR-GRSF1 abrogated the enhancement of hTERT protein induced by miR-138/346-loop mimics (Fig. 6i and Fig. S10). Next, we synthesized miR-138 mimics with the miR-346 mutant loop sequence (miR-138/miR-346-loop mut mimics) to determine whether GRSF1 plays a promoting role in a miR-346 sequence motif-dependent manner. Co-expression of Flag-GRSF1 and miR-138/miR-346-loop mut mimics abolished the translational enhancement induced by Flag-GRSF1 and miR-138/miR-346-loop mimics (Fig. 6j and Fig. S10). These data further confirmed that the role of GRSF1 in miRNA-346-mediated target genes is dependent upon the miR-346 “CCGCAU” motif.

### GRSF1 mediates the up-regulation of hTERT expression by miR-346 through recruiting hTERT transcripts to polyribosomes

**Figure 7:**
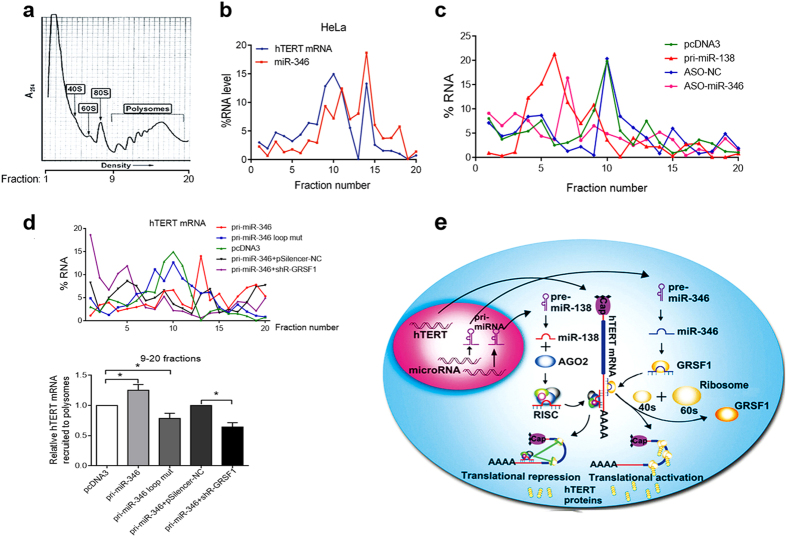
GRSF1 facilitates miR-346-mediated recruitment of hTERT mRNA to polyribosomes in a “bulge loop” motif-dependent manner. (**a**) The diagram shows ribosome footprints. 9–20 fractions represent the polysomes. (**b**) Ribosome purification was performed using a sucrose gradient sedimentation assay in mock HeLa cells. The distribution of miR-346 is similar to that of hTERT mRNA in the ribosomes. (**c**) In the ribosome footprinting assay, fraction 1–20 were collected from HeLa cells transfected with miR-138 and ASO-miR-346 and their respective control for the detection of hTERT mRNA by qRT-PCR. And the percentage of hTERT RNA in every fraction was deduced before plotting distribution curve. (**d**) The loop mutation reversed the right shift of hTERT mRNA induced by miR-346 (top panel) and reduced the recruitment of hTERT mRNA to the polyribosomes (bottom panel). GRSF1 depletion abolished the shift of hTERT mRNA to the right that was caused by miR-346 (top panel) and reduced the level of hTERT mRNA in the polyribosome fractions (9–20) as shown by qRT-PCR (bottom panel). All of the experiments were repeated at least three times. *p < 0.05. (**e**) A model depicting the roles of miR-346 and miR-138 in the regulation of hTERT expression. miR-346 and miR-138 competitively bind to a common site in the hTERT 3′UTR and facilitate the targeting of the hTERT mRNA to either the ribosome to promote translation by GRSF1 or to RISC to repress translation by AGO2, respectively.

Because GRSF1 augments the translation of mRNA by recruiting bound mRNAs to ribosomes^24^, we explored whether GRSF1 mediates the miR-346-dependent recruitment of hTERT mRNA to ribosomes. Sucrose gradient sedimentation assays were used to obtain ribosome fractions from HeLa cells, which are shown as ribosome profiles^31^ (Fig. 7a), and qRT-PCR was used to detect RNAs in each fraction. miR-346 was present in the ribosome fractions and had a similar distribution pattern as hTERT mRNA over the range of polyribosomes (Fig. 7b). Furthermore, miR-346 inhibition led to a left shift of hTERT mRNA distribution curve compared to the control group (Fig. 7c). However, Overexpression of miR-138 resulted in a left shift of hTERT mRNA distribution curve in ribosomes (Fig. 7c), indicating that miR-346 inhibition and miR-138 overexpression reduced hTERT translation. However, overexpression of the miR-346 loop mut abrogated the miR-346-mediated recruitment of hTERT transcripts to polyribosomes, and led to an approximately 64% reduction in hTERT mRNA bound to the polyribosomes (Fig. 7d), probablely due to the seed sequence of the miR-346 loop mut leading to hTERT downregulation. To determine whether miR-346-facilitated recruitment of hTERT transcripts to ribosomes depends on GRSF1, qRT-PCR showed that overexpression of miR-346 and knockdown of GRSF1 resulted in an approximately 35% decrease in the amount of hTERT mRNA in the polyribosome fractions compared with control group (Fig. 7d). Together, these results indicate that miR-346 promotes the recruitment of hTERT mRNA to ribosomes through GRSF1 to enhanced translation, while miR-138 may facilitate targeting hTERT mRNA to RISC to suppress translation (Fig. 7e).

## Discussion

Human cervical cancer is a leading disease in women worldwide, and hTERT is generally upregulated in this cancer^32^. hTERT plays a critical role in tumorigenesis and immortalized cells through its telomere-dependent and -independent activity^20^,^33^. The reactivation of hTERT expression is a critical step in carcinogenesis, which is required to maintain the rapid proliferation of cancer cells, and hTERT expression is strictly regulated in varying environments in human cells^20^,^23^. miRNAs are precise regulators that maintain cell homeostasis in various environments^4^,^6^. Among the miRNAs that are differentially expressed in cervical cancer, we focused on miR-346 and miR-138 and the mechanisms by which they regulate the hTERT transcript. In this report, we showed that miR-346, upregulated in cervical cancer tissues compared with adjacent normal tussues, promoted HeLa cell growth *in vitro* and HeLa cell-derived tumors *in vivo*, inferring its oncomiR role in cervical cancer. The fundamental function of miRNAs is to regulate their targets by affecting mRNA stability or suppressing translation. So we predicted a putative miR-346 binding site within hTERT 3′UTR and identified that miR-346 enhanced hTERT expression through directly targeting the hTERT 3′UTR, which may be related with the increased stability of hTERT mRNA. Moreover, a positive correlation between miR-346 and hTERT expression in cervical cancer tissues was also observed. In addition, depletion of hTERT suppressed the growth of HeLa cells *in vitro* and HeLa-derived tumors *in vivo*, which was similar to the phenotype of loss of miR-346, and ectopic expression of hTERT effectively rescued the suppression of HeLa cell growth caused by miR-346 inhibition. Collectively, these data suggest that miR-346 may upregulate hTERT expression to largely exert its cell growth-promotion effect in cervical cancer by specifically targeting the 3′UTR of the hTERT transcript.

miR-138 has been reported to repress hTERT expression in human anaplastic thyroid carcinoma cell lines^22^. Here, we found that miR-138 was downregulated in cervical cancer tissues compared with adjacent normal tissues and that miR-138 functioned as a tumor suppressor through suppressing hTERT expression by binding its 3′UTR in human cervical cancer lines. It has been reported that both miR-15a and miR-16 can bind to the same region of the BCL2 3′UTR to suppress its expression^16^. Our findings provide an example of two miRNAs that elicit opposing effects on a single gene by binding to a common site in its 3′UTR that coordinately regulates the expression of the target gene in cervical cancer. In detail, miR-346 and miR-138 target a common target region of the hTERT 3′UTR, nucleotides 20 to 34, and that the binding sites for both miRNAs overlap by 9 bases (Fig. 3a). hTERT mRNA levels correlate with the ratio of miR-346 to miR-138, and this ratio correlated with the rate of colony formation in HeLa cells. Furthermore, dot blot hybridization assays confirmed the competition between miR-346 and miR-138. Accordingly, miR-346 competes with miR-138 for binding to sites within the hTERT 3′UTR, resulting in enhanced the stability of hTERT mRNA and translation of the hTERT protein. These results indicate that miR-346 may not influence hTERT transcription, but that it protects hTERT mRNA from degradation, potentially contributing to the upregulation of hTERT expression. Thus, miR-346 may function as a decoy, while miR-138 may act as an inducer of decay to fine-tune hTERT levels by binding the common site in its 3′UTR.

In the most cases, miRNAs decrease protein production by either increasing mRNA degradation or suppressing mRNA translation through binding target 3′UTR^2^; however, the miRNA-mediated indirect upregulation of target gene can be modulated in response to different conditions in the same cells^1^,^8^,^9^. For example, miR-122-mediated repression of human CAT-1 mRNA occurs through the binding of HuR to AU-rich elements (AREs) in the CAT-1 3′UTR under stress conditions^34^, and miR-369-3p targets TNFα AREs to activate its expression in serum-starved conditions^11^. Moreover, some miRNAs have also been shown to induce gene activation directly. For instance, miR-10a interacts with the 5′UTR of mRNAs encoding ribosomal proteins to enhance their translation^35^. miR-346 targets the 5′UTR of the receptor-interacting protein 140 (RIP140) mRNA to upregulate its expression^36^. miR-1 has been also reported to represses translation in the cytoplasm, but positively enhances mitochondrial translation recently^37^. We also found that miR-20a and miR-490-3p upregulate TNKS2 and ERGIC3, respectively, by targeting their 3′UTRs^13^,^14^. In the present study, we found miR-346 targeted and enhanced the expression of hTERT through binding its 3′UTR. But the mechanisms are still unknown.

In order to address the above question, we first detected whether the miR-346-mediated upregulation of hTERT is dependent on AGO2, the key component of RISC. Here, we found that knockdown of AGO2 did not influence the miR-346-induced activation of the reporter or the endogenous hTERT mRNA and protein expression levels. These findings indicate that miR-346-mediated upregulation of hTERT occurs in an AGO2-independent manner through hTERT 3′UTR in human, although miR-346 has been also reported to upregulate RIP140 by targeting 5′UTR in an AGO2-independent manner in mice.

miRNA seed sequences are required for their function, but recent findings have demonstrated that sequences outside of the seed region may have important roles in miRNA function^1^. For example, a hexamer nucleotide in miR-29b is important for its nuclear import^38^, and a C-rich element in miR-328 endows the decoy activity through which miR-328 competes with hnRNP E2 for CREBA mRNA binding, resulting in the upregulation of CREBA^39^. Additionally, disruption of the miR-16 family through serum-induced cell cycle re-entry depends on both the seed and the 3′-end region sequences^40^. miR-1 stimulates the translation of the mitochondrial genes ND1 and COX1 through its seed and 3′ profile sequences in an AGO2-dependent and GW182-independent manner, respectively^37^. In this study, we found that the middle nt 8 to 13 (CCGCAU, 6 nt) of miR-346 do not match nt 28 to 24 of the hTERT 3′UTR and forms a “bulge loop” (miR-346 loop) when bound to the hTERT 3′UTR with RNAhybrid algorithm. As expected, mutation of the middle sequence abrogated the miR-346-mediated promotion of hTERT expression in HeLa and C33A cell lines and HeLa cell growth, indicating that miR-346 promotes hTERT expression in a miR-346 “CCGCAU” motif-dependent manner. The role of the motif was further confirmed by miR-346-mediated regulation of ACVR2B and SMAD3. miR-346 was predicted to form a “bulge loop” when bound to the ACVR2B 3′UTR, which was upregulated depending on the miR-346 loop, and not predicted to form a miR-346 loop when bound to the SMAD3 3′UTR, which was downregulated whether the miR-346 loop was mutated or not. Next, we synthesized a mutant miR-138 mimics whose middle sequence was replaced with that of wild type miR-346 (miR-138/346-loop mimics) and another mutant miR-138 mimics with the mutant loop sequence of miR-346 (miR-138/miR-346-loop mut mimics). Surprisingly, the miR-138/346-loop mimics upregulated hTERT expression compared to the downregulation observed in the presence of miR-138, and miR-138/miR-346-loop mut mimics abrogated the promotion of hTERT expression induced by the miR-138/346-loop mimics. These data illustrated that the miR-346 loop has significant effects on miR-346-mediated upregulation of target genes.

It has been reported that RNA binding proteins have the potential to regulate RNA expression. For example, Dnd1 prohibits miR-221-mediated downregulation of its target gene, p27, by binding to U-rich regions (URRs) within the p27 3′UTR^41^. GRSF1 can bind to influenza virus mRNAs through G-rich motif, such as AGGAU/AGGGT, and recruit them to polyribosomes to enhance translation, which take place in the cytoplasm^24^,^26^,^27^. However, the sites that it recognizes are diverse; for example, GRSF1 also binds to a 27-nt motif in the 5′UTR of the mitochondrial glutathione peroxidase 4 mRNA to enhance its expression^42^. Recent reports showed that GRSF1 also locates in the mitochondrial to process the newly formed RNA^28^,^29^. Here, GRSF1 was found to promote miR-346-mediated upregulation of hTERT, which was sequence-specific and required wild type miR-346 loop. Furthermore, RIP and RNA EMSA assays demonstrated that GRSF1 interacted with miR-346 or the miR-346/hTERT 3′UTR duplex and that mutation of the miR-346 middle “CCGCAU” motif abrogated this interaction. GRSF1 also mediated the promotion of miR-346 and miR-138/346-loop mimics on ACVR2B and hTERT, respectively, in which the miR-346 “CCGCAU” motif is also essential. Thus, our findings suggest that the miR-346 “CCGCAU” motif is a new GRSF1 binding motif and GRSF1 may be a universal mediator to upregulate gene expression. However, detailed studies are needed to determine the specific domain of GRSF1 that is responsible for binding the miR-346 “CCGCAU” motif.

To determine if the recruitment of the hTERT mRNA to polysomes was GRSF1-mediated and miR-346-dependent, we used a ribosome footprinting assay that has been widely used to identify translating mRNAs and associated proteins^31^,^43^, and found that hTERT mRNA levels in ribosomes positively correlated with miR-346 expression levels. However, miR-138 was not obviously observed in the ribosomes, and the hTERT mRNA levels in ribosomes negatively correlated with miR-138 expression, which may shift the hTERT mRNA to RISC, indicating that miR-346 can promote the recruitment of hTERT mRNA to ribosomes for translation. We found that the miR-346 loop mutant abrogated the ability of miR-346 to promote the recruitment of hTERT mRNA to ribosomes. Moreover, depletion of GRSF1, miR-346 reduced the enrichment of hTERT mRNA in polyribosomes. Thus, the miR-346 middle sequence is indispensable for GRSF1 as the mediator of miR-346-facilitated recruitment of hTERT mRNA to the ribosome. Together, our findings reveal a novel model where miR-346 enhances the expression of hTERT by binding to its 3′UTR and facilitating its recruitment to ribosomes in an AGO2-independent manner, while AGO2-associated miR-138 concomitantly suppresses hTERT expression (Fig. 7a).

In conclusion, miR-346-mediated upregulation and miR-138-mediated downregulation competitively coordinate the regulation of hTERT expression by binding to a common site in the hTERT 3′UTR, which promotes the growth of human cervical cancer cells. These opposing miRNA interactions not only add a new layer to the complexity of the mechanisms required for cervical cancer malignancy but also, more importantly, reveal new insights into the molecular mechanisms of miRNA action. Specifically, this work reveals a mechanism of miRNA-mediated gene activation where binding of a miRNA to a target mRNA 3′UTR along with the RNA binding protein GRSF1 facilitates the recruitment of the target gene mRNA to ribosomes for translation in a miRNA sequence motif-dependent manner. This regulatory mechanism suggests that multiple miRNAs have the ability to alter mRNA metabolism by targeting a common site in a 3′UTR and acting as molecular decoys or molecular decay signals to regulate mRNA translation.

## Methods

### Cell culture and transfection

The human cervical cancer cell lines HeLa or HeLa229 and C33A cells were maintained in RPMI 1640 (GIBCO) or MEMα (GIBCO), respectively, supplemented with 10% FBS, 100 IU/ml penicillin and 100 μg/ml streptomycin and incubated at 37 °C in a humidified chamber supplemented with 5% CO_2_. Transfections were performed with the Lipofectamine 2000 Reagent (Invitrogen) following the manufacturer’s protocols.

### Clinical tumor specimens

Eighteen pairs of cervical cancer samples and matched normal cervical tissues were obtained from the Cancer Center of Sun Yat-sen University of Medical Sciences. All of the samples were obtained with the patients’ informed consent and approved by the Ethics Committee of Sun Yat-sen University of Medical Sciences. The category of cervical samples was confirmed by pathological analysis. The methods were carried out in accordance with the approved guidelines. The large RNA and small RNA of tissue samples were isolated using a miRVana™ miRNA Isolation Kit (Ambion) following the manufacturer’s instructions. Detailed information of the characterization of each tumor specimen can be found in Table S2.

### Western blot assay

HeLa cell extracts were prepared and used for immunoblotting with RIPA lysis buffer. The following primary antibodies were used in the Western blot assay: (1) mouse monoclonal anti-hTERT (Tianjin Saier Biotech; 1:500), (2) rabbit anti-GAPDH (Tianjin Saier Biotech; 1:1000), (3) rabbit anti-GRSF1 (Tianjin Saier Biotech; 1:100), (4) mouse anti-Flag (MBL; 1:4000), (5) rabbit anti-ACVR2B and anti-SMAD3 (Tianjin Saier Biotech; 1:200). Anti-mouse and anti-rabbit IgG-HRP second antibodies were also used for western blotting. The signal was developed with enhanced chemiluminescence. Lab Works™ Image Acquisition and Analysis Software (UVP) were used for the quantification of the bands in the form of gray intensities. The gels have been run under the same experimental conditions. The full-length gels and blots are included in the supplementary information (Supplementary Fig. S8–10).

### *In vivo* tumor xenograft studies

Animal protocols were approved by Tianjin Medical University Animal Care and Use Committee. The methods were carried out in accordance with the approved guidelines. Six-week-old female BALB/c athymic nude mice (Institute of Zoology, Chinese Academy of Sciences) were used for the *in vivo* study. The animals were maintained under specific pathogen-free conditions. A total number of 1 × 10^6^ transfected HeLa cells were implanted subcutaneously into the armpit of nude mice. Seven mice were included in each group. Tumor weights were measured using an electronic scale, and the Student’s t-test was used to compare tumor growth among groups.

### RNA immunoprecipitation assay

RIP assay was carried out following the method described by Christoph Ufer^42^ with some modifications. Please refer the supplementary method for details.

### Assessing the stability of hTERT mRNA

A stock solution (5 mg/ml) of actinomycin D (ActD) was prepared in dimethyl sulfoxide (DMSO). ActD (5 μg/ml in culture medium) was added to HeLa cells after transfection with miRNAs and oligomers. qRT-PCR was used to detect the hTERT mRNA in HeLa cells harvested 0, 1 and 2 hours following the ActD treatment.

### Sucrose gradient sedimentation

Thirty five ml of 17%–57% continuous sucrose gradient in 50 mM NH_4_Cl, 50 mM Tris-Acetate at pH 7.0, 12 mM MgCl_2_, 150 mM NaCl, 100 μg/ml cycloheximide (add before use) and 1 mM DTT (add before use) was prepared one day prior to cell harvest. Transfected HeLa cells (10 million cells per flask) were washed with PBS, harvested and transferred to a 1.5 ml tube 30 hrs post transfection. Cells were resuspended in 1 ml of lysis buffer (20 mM HEPES-KOH, at pH 7.4, 15 mM MgCl_2_, 200 mM KCl, 100 μg/mL cycloheximide, 2 mM DTT, 500 U/mL RNase inhibitor and 0.1% NP-40). Then the cells were incubated in the buffer for 20 min at 4 °C, and the cell lysis was centrifuged at 12,000 rpm for 10 min at 4 °C to remove debris and nuclei. The supernatant was loaded to the top of the continuous sucrose gradient and centrifuged at 25,000 rpm for 4 hrs at 4 °C with SW28ti rotor (Beckman). After centrifugation, 1–20 fractions (1 ml per fraction) were collected from top to bottom of the gradient and digested with 100 mg proteinase K in 1% SDS and 10 mM EDTA for 30 min at 37 °C. RNAs were recovered by extraction with an equal volume of phenol-chloroform-isoamylalcohol, followed by ethanol precipitation overnight. After centrifugation (12,000 g, 30 min at 4 °C), pellets were washed twice with 80% ethanol, air-dried, and resuspended in 20 μl of RNase-free water. The amount of RNA was quantified by qRT-PCR.

### Equipment and settings

The photos from the colony formation assays and animal study were collected by Nikon D3200 (pixel dimension: 3020*1728, 8 bit) from October, 2011 to December, 2014 in Tianjin Life Science Research Center. EGFP and RFP intensity was tested using an F-4500 fluorescence spectrophotometer (HITACHI, Tokyo, Japan). EGFP intensity is normalized to RFP intensity. qRT-PCR was performed using the Bio-RAD™ iQ5 fluorescence quantitative PCR instrument. Lab Works™ Image Acquisition and Analysis Software (UVP) were used for the quantification of the western blot bands in the form of gray intensities (pixel dimension: 3020*1728, 8 bit, UVP, U.S.A.) from October, 2011 to January, 2015 in Tianjin Life Science Research Center. The Sucrose gradient sedimentation assay were performed with the Beckman ultracentrifugation LE-80 K with SW28ti rotor at 25,000 rpm for 4 hrs at 4 °C.

### Statistical analysis

All the data reported are representative of at least three independent experiments. The quantitative values were expressed as the mean ± SD. The hypothesis testing for significance between two groups was performed using Student’s *t* test, and the testing for significance between three or more groups was performed using one-way analysis of variance (ANOVA) followed by a Student-Newman-Keuls *q* test to compare each set of two groups with the software of GraphPad Prism 6.01. *p < 0.05, **p < 0.01, #: no statistical significance. The correlation between the expression levels of miR-346 or miR-138 and hTERT mRNA was calculated using the Pearson correlation coefficient with the software of GraphPad Prism 6.01.

## Additional Information

**How to cite this article**: Song, G. *et al.* miR-346 and miR-138 competitively regulate hTERT in GRSF1- and AGO2-dependent manners, respectively. *Sci. Rep.* **5**, 15793; doi: 10.1038/srep15793 (2015).

## Supplementary Material

Supplementary Information
