# Supplementary material for: miR-346 and miR-138 competitively regulate hTERT in GRSF1- and AGO2-dependent manners, respectively
Source: Sci Rep. 2015 Oct 28;5:15793. doi: 10.1038/srep15793 (PMC4623477; doi:10.1038/srep15793)
Supplement: Supplementary Information [file srep15793-s1.pdf]

Supplementary Information

miR-346 and miR-138 competitively regulate hTERT in GRSF1- and AGO2-dependent manners, respectively

Ge Song#, Renjie Wang#, Junfei Guo#, Xuyuan Liu#, Fang Wang, Ying Qi, Haiying Wan, Min Liu, Xin Li, Hua Tang*

#These authors contributed equally to this work

*Corresponding author: Hua Tang

Figures S1-S10

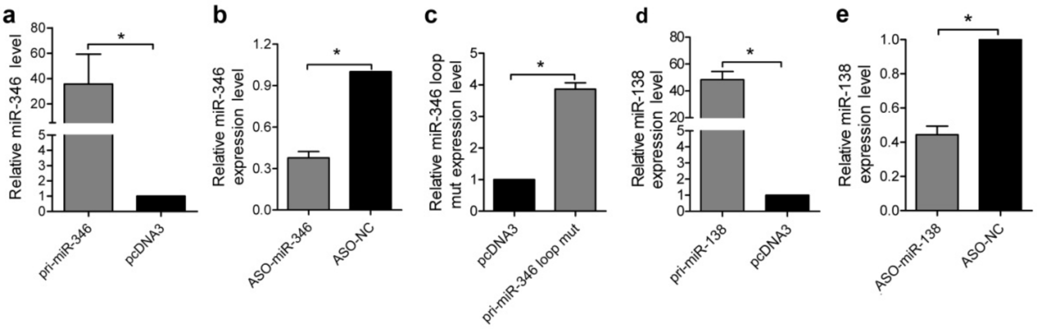

Figure S1. Validation of efficiency of plasmids and oligomers in transfected HeLa cells. (a-e) Small RNA was extracted from HeLa cells transfected with ASO-miR-346 or ASO-miR-138 and control oligomers; pri-miR-346, pri-miR-346 loop mut (Note: used different forward primers in Table S1, 346 Frd and 346 mut Frd to amplify miR-346, miR-346 loop mut, respectively) or pri-miR-138 and pcDNA3 as the control vector, and the expression of miR-346 and miR-138 was measured by qRT-PCR. U6 snRNA was regarded as the endogenous normalizer and the relative miR-346 and miR-138 expression levels (mean ± SD) were shown (* $p < 0.05$).

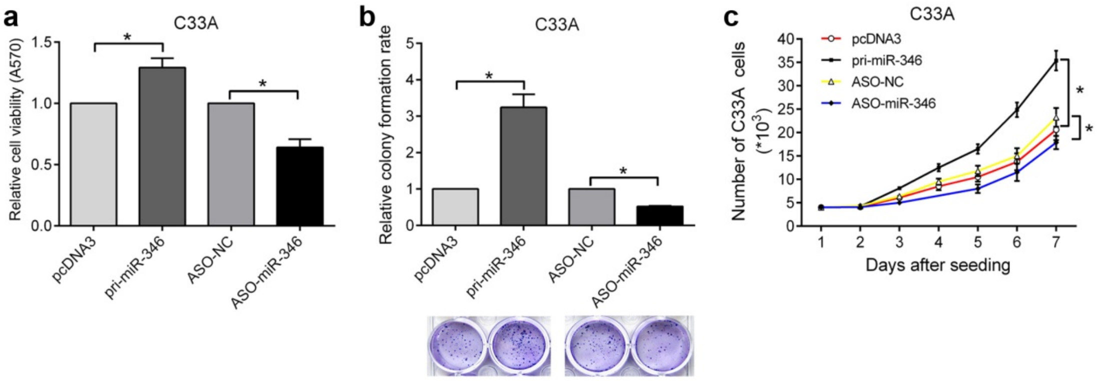

Figure S2. miR-346 promotes the growth of human cervical C33A cells *in vitro*.

(a-c) Ectopic expression of miR-346 and inhibition of miR-346 with ASOs affect the cellular viability and growth capacity of the human cervical cancer cell line C33A as determined by MTT assays (a), colony formation assays (b) and growth curve assays (c). Significant differences were identified by Student’s t test (*p < 0.05). The error bars indicate SD (n = 3).

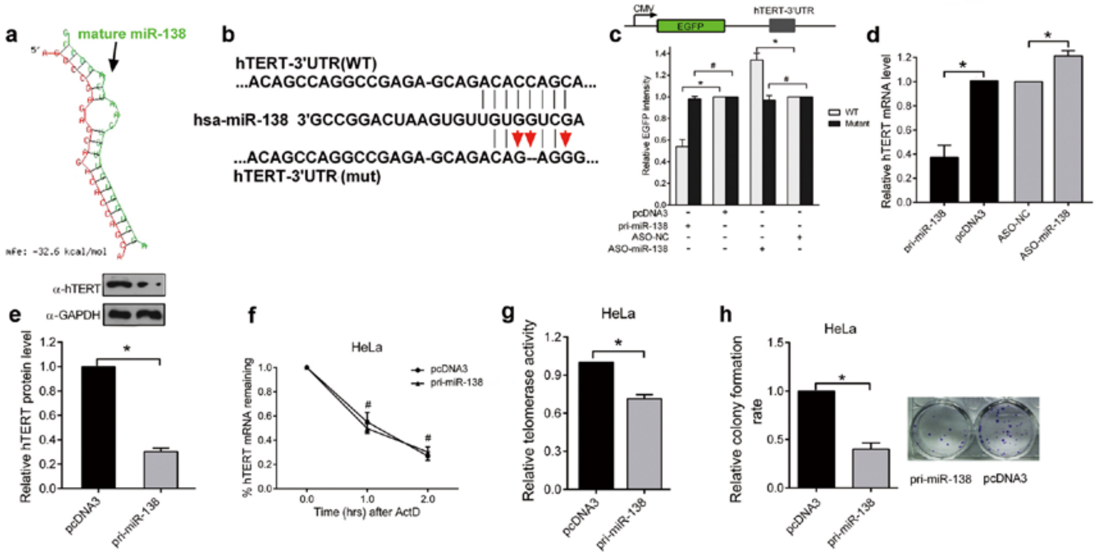

Figure S3. miR-138 targets the hTERT transcript and down-regulates its expression.

(a) miR-138 can potentially form a strong secondary structure with the target sequence of the hTERT 3’UTR, as predicted by RNAHybrid. mfe = -32.6 kcal/mol. (b) The predicted miR-138 binding site on the hTERT mRNA 3’UTR and the point mutation of the "seed

region" binding site on the hTERT mRNA 3'UTR are shown. (c) An EGFP reporter assay was performed to determine the effect of miR-138 on the hTERT 3'UTR. HeLa cells were transfected with the wild type EGFP-hTERT 3'UTR (WT) or mutated EGFP-hTERT 3'UTR (Mutant) reporter vector and ASO-miR-138, pri-miR-138, or their respective controls. (d and e) The influences of miR-138 on hTERT expression were evaluated by qRT-PCR (d) and western blot (e). The levels of hTERT mRNA and protein were normalized to the levels of β-actin mRNA and GAPDH protein, respectively. The blot was cropped and the full-length blot is presented in Supplementary Figure S8. (f) miR-138 did not affect the stability of hTERT mRNA. The remaining hTERT mRNA levels at 0, 1 and 2 hr after ActD treatment (5 μg/ml) of HeLa cells were measured by qRT-PCR and showed no significant difference between miR-138 and control groups. (g and h) Evaluation of the telomerase activity and growth capacity of HeLa cells transfected with pri-miR-138 and control plasmid as determined by the *T*elo*TAGGG* Telomerase PCR ELISA^{PLUS} kit (Roche) (g) and colony formation assays (h). All the experiments were performed in triplicate. The results are presented as means ± SD, *p < 0.05. #p > 0.05.

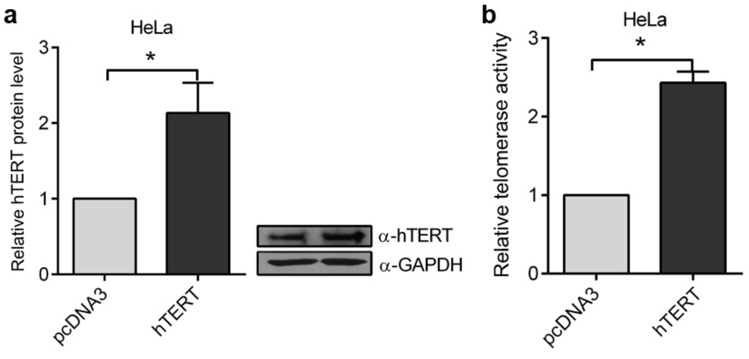

Figure S4. Evaluation of the expression level of hTERT protein and telomerase activity.

(a) Western blot evaluation of the efficiency of expression from the hTERT expression vector

with a myc tag. GAPDH was used for normalization. The blot was cropped and the full-length blot is presented in Supplementary Figure S10. (b) Quantification of telomerase activity using the *T*elo*TAGGG* Telomerase PCR ELISA^{PLUS} kit (Roche) in cells transfected with the expression vector (hTERT) or control vector (pcDNA3). *$p < 0.05$.

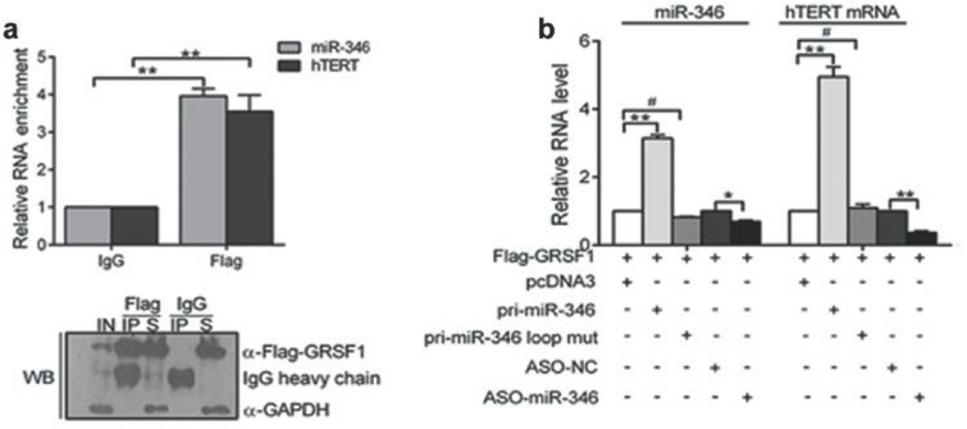

Figure S5. GRSF1 interacts with miR-346/hTERT mRNA in a “CCGCAU” motif dependent manner.

(a) The levels of hTERT mRNA and miR-346 that immunoprecipitated with the Flag-GRSF1 complex were detected by qRT-PCR. The bottom panel shows the efficiency of the immunoprecipitation. S: supernatant. Unsaturated anti-Flag antibody resulted in residues in the supernatant. The blot was cropped and the full-length blot is presented in Supplementary Figure S10. (b) The levels of hTERT mRNA in the GRSF1 immunoprecipitation complex correlated well with the levels of miR-346 in HeLa cells co-transfected with Flag-GRSF1 and pri-miR-346, pri-miR-346 loop mut, or control vectors as well as ASO-miR-346 or ASO-NC. **$p < 0.01$, *$p < 0.05$, #$p > 0.05$.

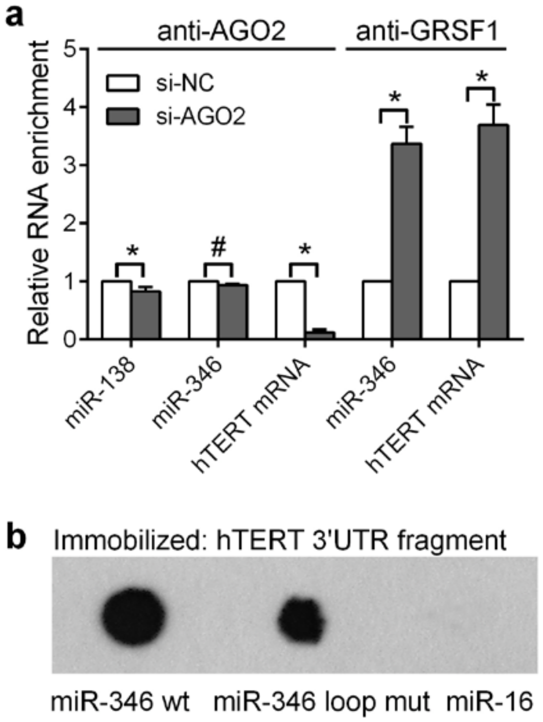

Figure S6. The interaction between miR-138/miR-346 and hTERT mRNA upon AGO2 depletion and the interaction between miR-346 loop mut and hTERT 3'UTR fragment.

(a) RNA IP and qRT-PCR assays were performed to detect the enriched levels of miR-138/hTERT mRNA or miR-346/hTERT mRNA in AGO2 or GRSF1 complexes, respectively, with HeLa cells transfected with si-AGO2 and negative control. AGO2 antibody (rabbit) and GRSF1 (mouse) antibody were used in the RIP assay. (b) The dot blot hybridization assay was performed to determine the interaction between miR-346 loop mut and hTERT UTR fragment. And miR-346 wt and miR-16 were used as a positive and a negative control, respectively. The probes were biotin-labled miR-346 wt, miR-346 loop mut and miR-16. The blot represented the full-length blot. *$p < 0.05$, #$p > 0.05$.

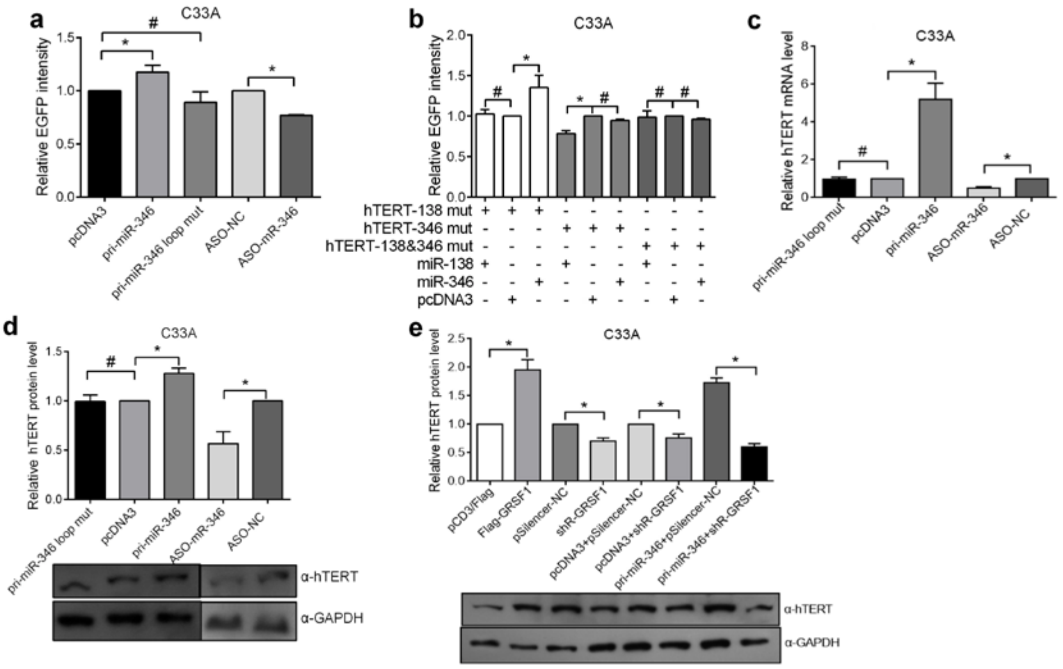

Figure S7. miR-346 upregulates hTERT in a GRSF1-dependent manner.

(a and b) The EGFP reporter assays were performed with wild type and the three types of mutant hTERT mRNA 3’UTR presented in Fig. 3a in C33A cells transfected with pri-miR-346, pri-miR-138, pri-miR-346 loop mut and ASO-miR-346 as well as their respective controls. (c and d) qRT-PCR and Western blot assays were used to illustrate the effects of wt/loop mutant miR-346 and ASO-miR-346 on endogenous hTERT expression. β-actin and GAPDH serve as loading controls of RNA and protein. (e) Western blot assay was conducted to demonstrate whether GRSF1 influences miR-346-mediated hTERT expression. The blots were cropped and the full-length blots are presented in Supplementary Figure S10. $^{**}p < 0.01$, $^{*}p < 0.05$, $^{\#}p > 0.05$.

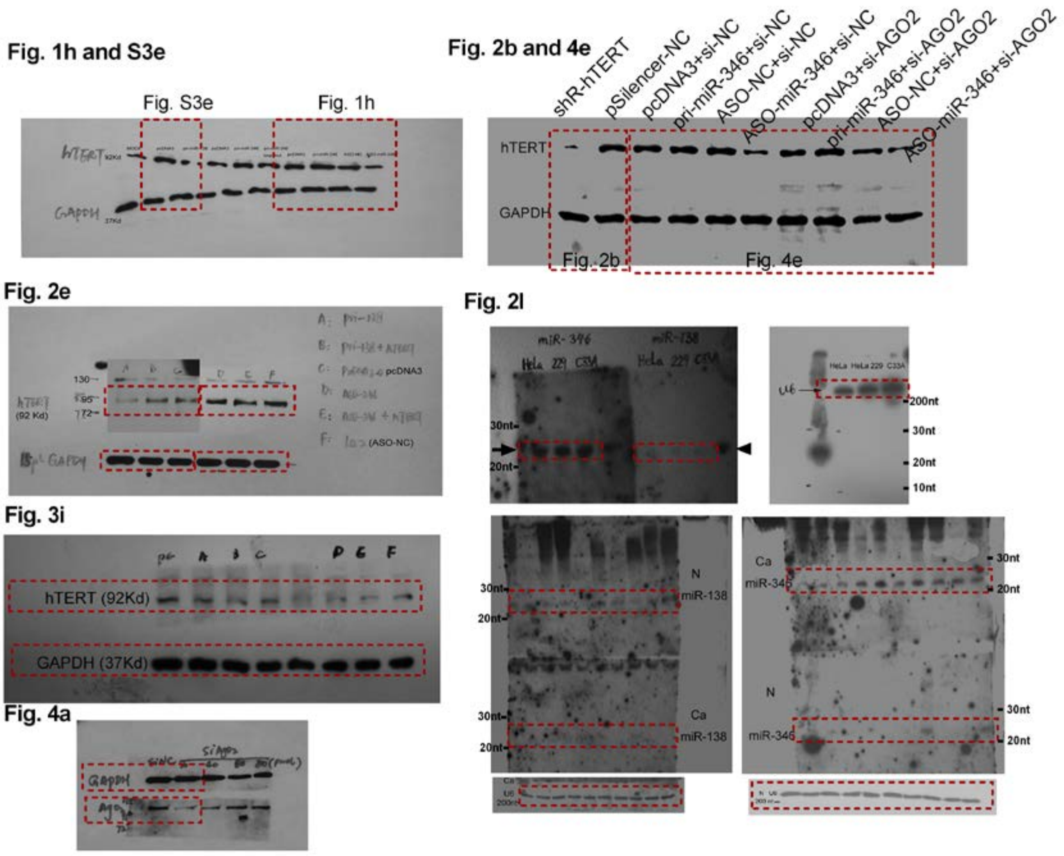

Figure S8. Uncropped, unprocessed images of blots and gels.

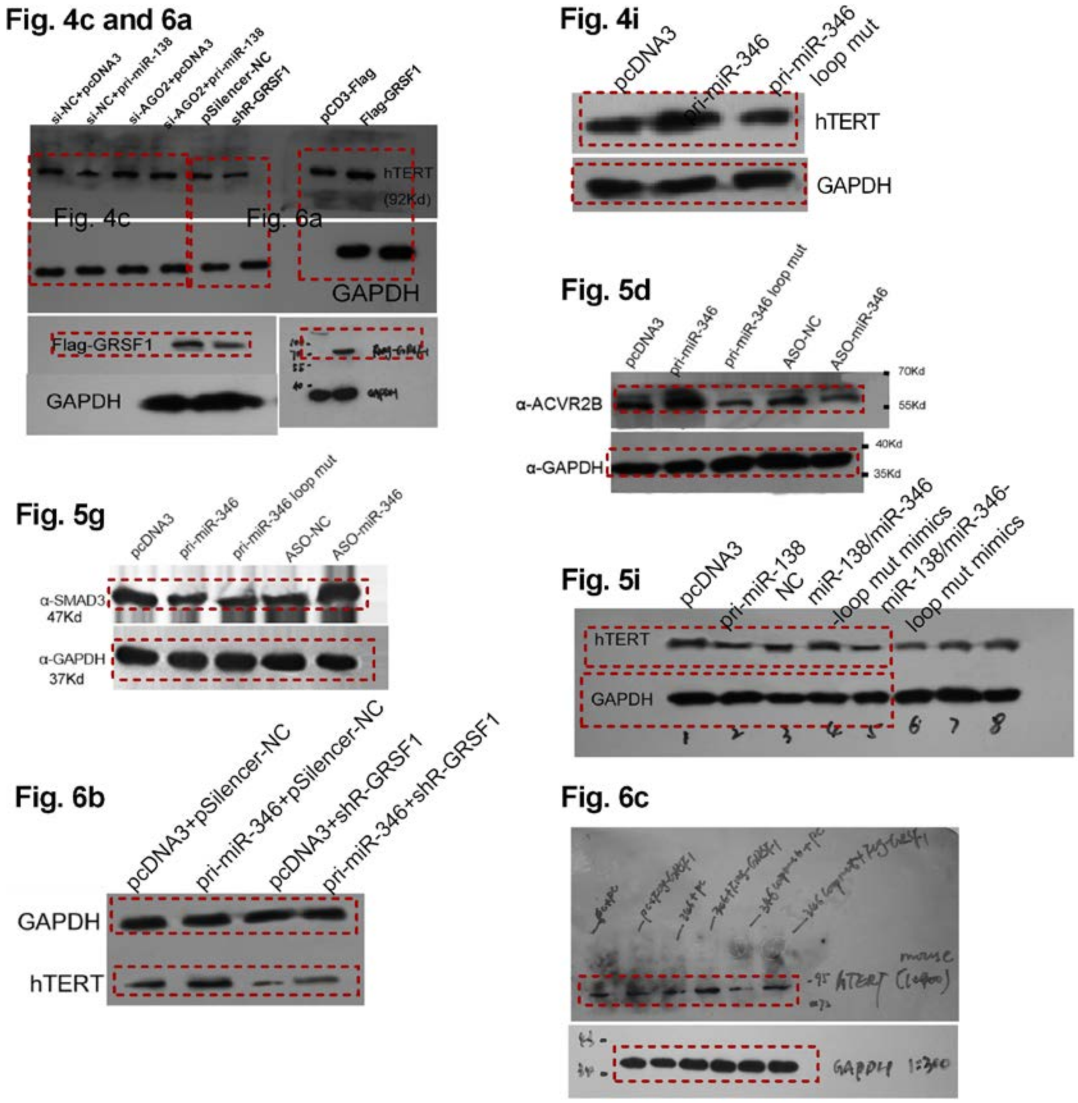

Figure S9. Uncropped, unprocessed images of blots and gels.

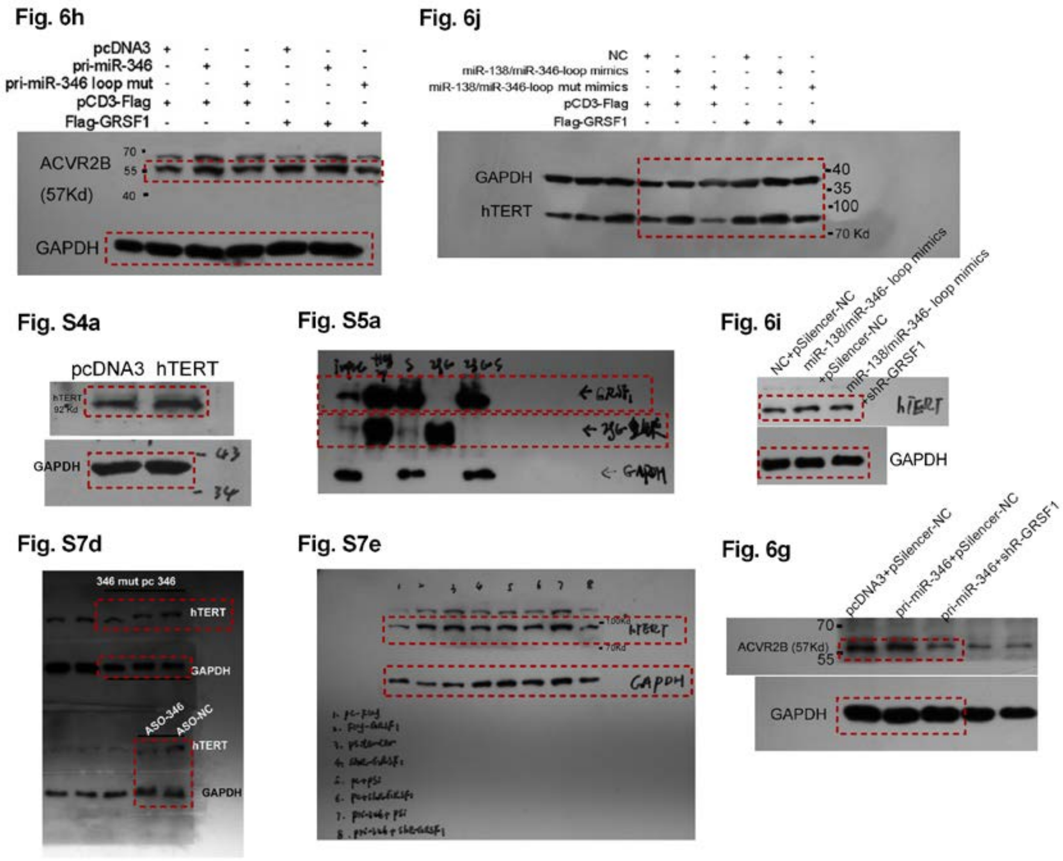

Figure S10. Uncropped, unprocessed images of blots and gels.

Tables S1-S2

Table S1. Primer sequences and oligomers used in this study

Oligo name	Sequence
pri-346-S	5’ CACGGATCCCTTGTCAGCAAGGAGTG 3’
pri-346-AS	5’ CGGAATTCTAGGTTGGGAGCGAAGTG 3’
pri-138-S	5’ CGTGGATCCGAAGGCAGTGAAATGTTAGC 3’
pri-138-AS	5’ CCAGAATTCCTCTGTGACGGGTGTAGCTG 3’
ASO-miR-346/miR-346 probe	5’ AGAGGCAGGCATGCGGGCAGACA 3’/ 5’ UGUCUGCCCGCAUGCCUGCCUCU 3’
miR-16 probe	5’ UAGCAGCACGUAAAUAUUGGCG 3’
ASO-miR-138/miR-138 probe	5’ CGGCCTGATTCACAACACCAGCT 3’ / 5’ AGCUGGUGUUGUGAAUCAGGCCG 3’
U6 probe	5’ TGTGCTGCCGAAGCGAGCAC 3’
hTERT-346-UTR-S	5’ TGCGGATCCTCAGGACAGCCCAGACG 3’
hTERT-346-UTR-AS	5’ CCTGAATTCCTCGGCCAAACACTCACTC 3’
hTERT-346-UTR-MS	5’ CAGGCCGAGAGGGCGCACCAGCAGCA 3’
hTERT-346-UTR-MA	5’ TGCTGCTGGTGCGCCCTCTCGGCCTG 3’
hTERT-138-UTR-MS	5’ GAGAGCAGACAGAGGAGCCCTGTCACG 3’
hTERT-138-UTR-MA	5’ CGTGACAGGGCTCCTCTGTCTGCTCTC 3’
hTERT-346 & 138-UTR-MS	5’ CAGGCCGAGAGGGCGCAGAGGGCGAGCCCTGTCACG 3’
hTERT-346 & 138-UTR-MA	5’ CGTGACAGGGCTCGCCCTCTGCGCCCTCTCGGCCTG 3’
ACVR2B-346-UTR-Top	5’ GATCCTGGCCTAAAGCAGACATCCATGTAAGCTTG 3’
ACVR2B-346-UTR-Bot	5’ AATTCAAGCTTACATGGATGTCTGCTTTAGGCCAG 3’
SMAD3-346-UTR-Top	5’ GATCCAGGCTGCTGGCAGACGTCTCCAAGCTTG 3’
SMAD3-346-UTR-Bot	5’ AATTCAAGCTTGGAGACGTCTGCCAGCAGCCTG 3’
hTERT-qRT-forward	5’ GCGGAAGACAGTGGTGAACT 3’
hTERT-qRT-reverse	5’ AGCTGGAGTAGTCGCTCTGC 3’
hTERT-CDS-S	5’ AAGAATTCATGCCGCGCGCTCCCCGC 3’
hTERT-CDS-AS	5’ GCCGGTCTAGATCAGTCCAGGATGGTCTTG 3’
miR-346-RT	5’ GTCGTATCCAGTGCAGGGTCCGAGGTGCACTGGATACGACAGAGGCA 3’
miR-138-RT	5’ GTCGTATCCAGTGCAGGGTCCGAGGTGCACTGGATACGACCGGCCTG 3’
U6-RT	5’ GTCGTATCCAGTGCAGGGTCCGAGGTATTCGCACTGGATACGACAAAATATGGAAC 3’
346-Fwd	5’ TGCGGTGTCTGCCCGCATGCCTG 3’
346 mut Fwd	5’ TGCGGTGTCTGCAAAATA 3’
138-Fwd	5’ TGCGGAGCTGGTGTTGTGAATC 3’
U6-Fwd	5’ TGCGGGTGCTCGCTTCGGCAGC 3’
Reverse	5’ CCAGTGCAGGGTCCGAGGT 3’
shR-hTERT-Top	5’ AGCAAGUUGCAAAGCAUUGGAAUTT 3’
shR-hTERT-Bottom	5’ AUUCCAAUGCUUUGCAACUUGCUTT 3’
hTERT-S	5’ GCCGAATTCGCCACCATGCCGCGCGCTCCCCGCTGC 3’
hTERT-AS	5’ GTCCGCTCGAGGTCCAGGATGGTCTTGAAGTC 3’
si-AGO2-Top	5’ GCACGGAAGUCCAUCUGAATT 3’

si-AGO2-Bottom	5’ UUCAGAUGGACUUCCGUGCTT 3’
hTERT 3’UTR	5’ GCCCACAGCCAGGCCGAGAGCAGACACCAGCAGCCCUG 3’
hTERT 3’UTR-138 mut	5’ GCCCACAGCCAGGCCGAGAGCAGACAGAGGAGCCCUG 3’
hTERT 3’UTR-346 mut	5’ GCCCACAGCCAGGCCGAGAGGGCGCACCAGCAGCCCUG 3’
hTERT 3’UTR-138&346 mut	5’ GCCCACAGCCAGGCCGAGAGGGCGCAGAGGAGCCCUG 3’
miR-346	5’ UGUCUGCCCGCAUGCCUGCCUCU 3’
miR-138	5’ AGCUGGUGUUGUGAAUCAGGCCG 3’
miR-138/miR-346-loop mimics	5’ AGCUGGUGUUGUGCCGCAUGGCCG 3’
miR-138/miR-346 loop mut mimics	5’ AGCUGGUGUUGUGAAAAUAGGCCG 3’
3’-biotin-conjugated miR-346	5’ UGUCUGCCCGCAUGCCUGCCUCU 3’
3’-biotin-conjugated miR-346 loop mut	5’ UGUCUGCAAAATAGCCUGCCUCU 3’
3’-biotin-conjugated hTERT	5’ GCCCACAGCCAGGCCGAGAGCAGACACCAGCAGCCCUG 3’
GRSF1-S	5’ ACGGAATTCGAGTCCATGGCCGGCACGCGC 3’
GRSF1-AS	5’ GCAGCACGCGAGGCTTTTCCTTTTGGACATGAGTTCAGGAAC 3’
GRSF1-Top	5’GATCCATGAGGATATTCAACCCATGACACTCGAGTGTCATGGGTTGAATATCCTCATTTTTTGA 3’
GRSF1-Bottom	5’AGCTTCAAAAAATGAGGATATTCAACCCATGACACTCGAGTGTCATGGGTTGAATATCCTCATG 3’

Table S2. Detail information of the cervical tissues

Tissue number	Age	Pathology typing	Differentiation (G1, G2, G3)	Lymph node metastasis
1375	48	squamous cell cancer	G3	Yes
1392	37	squamous cell cancer	G3	Yes
1451	45	squamous cell cancer	G3	Yes
1466	46	squamous cell cancer	G3	Yes
1522	46	squamous cell cancer	G2	Yes
1557	48	squamous cell cancer	G3	Yes
1578	47	squamous cell cancer	G3	Yes
1428	53	squamous cell cancer	G2	Yes
1580	43	squamous cell cancer	G3	No
1582	42	squamous cell cancer	G2	Yes
1587	45	squamous cell cancer	G3	No
1588	54	squamous cell cancer	G2	Yes
1592	46	squamous cell cancer	G2	No
1612	38	squamous cell cancer	G3	Yes

1616	53	adenocarcinoma	G3	Yes
1617	50	squamous cell cancer	G3	No
1625	54	squamous cell cancer	G3	No
1626	47	squamous cell cancer	G3	No

Supplementary methods

Oligonucleotides and plasmids

The primers used to construct the pri-miRNA, hTERT, ACVR2B and SMAD3 mRNA 3'UTRs, hTERT and GRSF1 overexpression vectors and shR-hTERT and shR-GRSF1 plasmids were obtained from the Beijing AuGCT Inc, China. 2-O'-methyl- and 3'-amino-C6-modified anti-miRNA oligomers, miR-138/miR-346-loop mimics, miR-138/miR-346-loop mut mimics, siRNA-AGO2 and biotin-conjugated or unconjugated miR-346, miR-346 loop mut and hTERT mRNA fragments (38 nts) were purchased from Genepharma (Shanghai, China). Details of the primer sequences and oligomers can be found in Table S1.

Cell viability, colony formation and growth curve assays

For the MTT assay, cells were seeded in a 96-well plate (in triplicate) at a concentration of 8,000 cells per well the day before the transfection. Cells were first transfected with different plasmids and their respective controls. Forty-eight hr after transfection, the absorbance of the viable, proliferating cells was measured at 570 nm using a μQuant Universal Microplate Spectrophotometer (Bio-tek Instruments). For the colony formation assay, the transfected cells were counted and seeded in 12-well plates (in triplicate) at a concentration of approximately 200 cells per well. Fresh culture medium was replaced every three days. On

the 10th day after seeding, the cells were stained using crystal violet and colonies with more than 50 cells were counted. The rate of colony formation was calculated using the following equation: colony formation rate = (number of colonies / number of seeded cells) × 100%. The growth curve assay was performed by seeding 4,000 transfected cells in 24-well plates and counting the HeLa cells and the C33A cells daily for 8 and 7 days, respectively, after seeding.

miRNA target prediction

miRNA targets were predicted using the TargetScan (http://www.targetscan.org/) and MicroCosm Targets (http://www.ebi.ac.uk/enright-srv/microcosm/htdocs/targets/v5/) algorithms. The secondary structure of microRNAs and target gene 3'UTRs was analyzed using RNAHybrid algorithm (http://bibiserv.Techfak.uni-Bielefeld.de/rnahybrid/).

EGFP reporter assay

The EGFP reporter assay was performed as previously described[1]. In brief, the 3'-untranslated region of hTERT mRNA or a series of hTERT mRNA 3'UTR mutants were amplified by PCR using the primers shown in Table S1. The PCR products were then cloned into the pcDNA3/EGFP vector at the BamHI and EcoRI sites downstream of the EGFP coding region. The ACVR2B and SMAD3 mRNA 3'UTRs were annealed using the primers listed in Table S1 and cloned into the same vector mentioned above. HeLa cells were co-transfected with a reporter plasmid and either pri-miR-346, pri-miR-138 or the pcDNA3 control vector in 48-well plates. The pDsRed2-N1 vector (Clontech) expressing RFP was spiked in to normalize the transfection. After 48 hours, the cells were lysed and the fluorescence

intensities of EGFP and RFP were detected with a F-4500 fluorescence spectrophotometer (HITACHI).

RNA extraction and qRT-PCR

At indicated time points post transfection or post other treatments, total RNAs were extracted from cells using the TRIzol reagent (Invitrogen). Expression of mature miRNAs was determined using miRNA-specific stem-loop qRT-PCR[2,3] in an iQ5 Real-Time PCR Detection System (Bio-Rad) with the SYBR Premix Ex Taq™ Kit (TaKaRa). U6 snRNA was used for normalization. The following PCR program was used to detect mature miR-346 and miR-138: (1) 95°C for 3 min, and (2) 40 cycles of 95°C for 30 s, 56°C for 30 s, and 72°C for 30 s. For hTERT, ACVR2B and SMAD3 mRNA detection, the following PCR conditions were used: (1) 95°C for 5 min and (2) 40 cycles of 95°C for 30 s, 58°C for 30 s, and 72°C for 40 s. β-actin was used as an internal control.

Telomerase activity detection

The activity and quantification of telomerase in the parental and transfected cervical cancer cells was detected using the *T*elo*TAGGG* Telomerase PCR ELISA^{PLUS} kit (Roche) following the manufacturer's instructions.

Northern blot assay

Northern blot assay was performed as previously described[4]. Small RNAs were separated by electrophoresis on 20% PAGE-urea gels. The RNA was blotted and cross-linked onto nylon

membranes (Ambion) and hybridized with γ-^{32}P end-labeled (Furui Company, Beijing, China) ASO-miR-346, 138 and U6 snRNA, respectively (sequences can be found in Table S1). All of the radioisotopes were imaged at −80°C.

RNA immunoprecipitation assay

RIP assay was carried out following the method described by Christoph Ufer[5] with some modifications. HeLa cells transfected with Flag-GRSF1 were trypsinized, resuspended in chilled PBS, and centrifuged at 5,000 rpm for 5 minutes at 4°C 24 hours post transfection. The pellets were resuspended in lysis buffer [50 mM Tris-HCl, PH 7.5, 1% NP-40, 0.5% sodium deoxycholate, 1 mM EDTA, 140 mM NaCl, 1.5 mM $MgCL_2$, 1 mM DTT, 100 U/ml RNasin, and 1x proteinase inhibitor cocktail (Roche)], incubated on ice for 10 minutes with occasional flicking, and then centrifuged at 16,000g at 4°C for 5 minutes. The supernatant was transferred to a new 1.5 ml tube. The samples were pre-cleaned by adding 50 µl of protein G slurry to each tube and rotating the samples at 4°C for 2 hours. After centrifugation at 5,000 g for 5 minutes at 4°C, the supernatant was transferred to a new tube. Ten µg of anti-Flag antibody was added to the supernatants; the same amount of mouse IgG was added to the non-specific control sample. After a rotation at 4°C overnight, 50 µl of protein G slurry was added to the supernatants and the samples were rotated at 4°C for 2 more hours. Following centrifugation at 5,000 g for 5 minutes at 4°C, the supernatants were discarded and the samples were resuspended in 1 ml lysis buffer. The samples were centrifuged at 5,000 g for 5 minutes at 4°C and then the supernatants were discussed. This step was then repeated. The pellet was then washed twice with high salt lysis buffer (the same as the lysis buffer

except that NaCl is at 700 mM). The RNAs in the pellets were released from the samples after proteinase K treatment for 30 minutes at 50°C, extracted with phenol: chloroform: isoamyl alcohol (25: 24: 1), and then precipitated in alcohol overnight. The RNAs were then quantified by qRT-PCR.

Dot blot hybridization assay

RNA dot hybridization was performed as described previously[6] with some modification. In brief, we first synthesized the hTERT mRNA 3'UTR containing miR-346 and miR-138 binding sites (hTERT 138-346 UTR, miR-346, and miR-138). For dot hybridization, we denatured hTERT mRNA 3'UTRs in RNA denaturing buffer at 65°C for 5 minutes. After incubating on ice for 2 minutes, the RNA was diluted with an equal volume of 20 x SSC buffer to a final concentration of 0.02 μg/μl. One μl of the denatured RNA was applied to a nylon membrane (Ambion) and pre-treated with 20 x SSC for at least 1 hour. The samples were air dried and then UV-crosslinked with a CL-1000 Ultraviolet Crosslinker. The membranes were then sealed in plastic bags containing the pre-hybridization buffer (6 x SSC, 10 x Denhardt's solution and 1% SDS) and rotated at 65°C for 3 hours in a Fisher Biotech hybridization incubator. The membrane was transferred to a new plastic bag containing hybridization buffer (6 x SSC, 10 x Denhardt's solution, 0.2% SDS, and 5 mg/ml sodium pyrophosphate) supplemented with either γ-^{32}P end-labeled miR-346 or miR-138 probe (final concentration: 0.004 μg/μl), respectively, and rotated at 65°C overnight. Next day, the membranes were washed twice with buffer I (2 x SSC and 0.1% SDS) at room temperature, for 5 minutes each time, and then washed twice with washing buffer II (0.1 x SSC and 0.1%

SDS) at 55°C, 5 minutes each time. The membranes were then exposured at -80°C for 12 to 24 hours. For the competition assay, 5-, 15-, and 45-fold excess cold miR-346 or miR-138 probe were added to the reactions.

RNA electrophoretic mobility shift assay

Single stranded 3'-biotin-conjugated miR-346 (B-miR-346), miR-346-loop mut (B-miR-346 mut), the complementary hTERT mRNA 3'UTR sequence (38 nts) (B-hTERT 3'UTR), and the corresponding unconjugated oligonucleotides were purchased from the GenePharma Company (Shanghai, China). First, the biotin-conjugated miR-346 and miR-346 loop mut or unconjugated ones were annealed with the unconjugated hTERT mRNA in 5 x RNA annealing buffer[7] (30 mM HEPES-KOH, pH 7.4, 100 mM KCl, 2 mM $MgCl_2$, and 50 mM NH_4Ac). The annealing samples were incubated for 1 minute at 90°C and slowly cooled to room temperature. B-miR-346, B-miR-346-loop mut, B-hTERT 3'UTR or the annealed duplexes (B-miR-346/hTERT and B-miR-346 mut/hTERT) (25 nM) were incubated with 6.0 µg of HeLa cell extract[7] overexpressing Flag-GRSF1 in the absence or presence of a 200-fold excess of unconjugated competitors or 1.5 µg of anti-Flag antibody in 1x EMSA binding buffer (Pierce, 20158) for 30 min at room temperature. The RNA-protein complex was resolved on a 6% non-denaturing polyacrylamide gel in 0.5 x TBE buffer. The complex was transferred to a nylon membrane in 0.5x TBE buffer at 350 mA for 30 min. The membrane was then treated according to the manufacturer's instructions [LightShift Chemiluminescent RNA EMSA kit (Pierce), 20158].

Supplementary references

1 Yan, Y. *et al.* MicroRNA-10a is involved in the metastatic process by regulating Eph tyrosine kinase receptor A4-mediated epithelial-mesenchymal transition and adhesion in hepatoma cells. *Hepatology* **57**, 667-677 (2013).

2 Chen, C. *et al.* Real-time quantification of microRNAs by stem-loop RT-PCR. *Nucleic Acids Res* **33**, e179 (2005).

3 Liu, T., Tang, H., Lang, Y., Liu, M. & Li, X. MicroRNA-27a functions as an oncogene in gastric adenocarcinoma by targeting prohibitin. *Cancer letters* **273**, 233-242 (2009).

4 Pall, G. S., Codony-Servat, C., Byrne, J., Ritchie, L. & Hamilton, A. Carbodiimide-mediated cross-linking of RNA to nylon membranes improves the detection of siRNA, miRNA and piRNA by northern blot. *Nucleic Acids Res* **35**, e60 (2007).

5 Ufer, C. *et al.* Translational regulation of glutathione peroxidase 4 expression through guanine-rich sequence-binding factor 1 is essential for embryonic brain development. *Genes Dev* **22**, 1838-1850 (2008).

6 Faivre-Sarrailh, C., Ferraz, C., Liautard, J. P. & Rabie, A. Effect of thyroid deficiency on actin mRNA content in the developing rat cerebellum. *International journal of developmental neuroscience : the official journal of the International Society for Developmental Neuroscience* **8**, 99-106 (1990).

7 Deschenes-Furry, J. *et al.* The RNA-binding protein HuR binds to acetylcholinesterase transcripts and regulates their expression in differentiating skeletal muscle cells. *J Biol Chem* **280**, 25361-25368 (2005).
